# Development of photodynamic therapy in treating oral diseases

**DOI:** 10.3389/froh.2024.1506407

**Published:** 2025-01-15

**Authors:** Ling Wang, Qiang Chen, Dan Liu

**Affiliations:** ^1^ Department of Stomatology, Hospital of Chengdu Office of People’s Government of Tibetan Autonomous Region (Hospital.C.T.), Chengdu, Sichuan, China; ^2^ Department of Stomatology, The Traditional Chinese Medicine Hospital of Longquanyi, Chengdu, Sichuan, China; ^3^ Department of Stomatology, Ren Ai Community Healthcare Center of Longquanyi District, Chengdu, Sichuan, China

**Keywords:** photodynamic therapy, oral oncology, periodontitis, caries, peri-implant infection(s), candidiasis

## Abstract

The morbidity of oral disorders, including gingivitis, caries, endodontic-periodontal diseases, and oral cancer, is relatively high globally. Pathogenic cells are the root cause of many oral disorders, and oral therapies depend on eradicating them. Photodynamic therapy (PDT) has been established as a potential and non-invasive local adjuvant treatment for oral disorders. PDT consists of three essential components: photosensitizer (PS), a light source with a certain wavelength, and oxygen dissolved in the cells. These three components can interact to cause damage to proteins, lipids, nucleic acids, and other biological components within diseased tissues. Herein, we aimed to provide a detailed understanding of PDT and how it can treat oral diseases. Concerns about PDT and potential remedies are also a factor. PDT has been shown in numerous clinical studies to be an efficient supplementary therapy that can reduce pathogenic cells. The PDT has great potential for dental applications, including treating bacterial and fungal infections during root canal therapy and preventing oral cancer, potentially malignant disorders, periodontitis, dental caries, and peri-implant disorders. Although PDT has been promoted as having significant potential and utility in dentistry, more clinical research must be conducted before being used broadly.

## 1 Introduction

Millions of individuals worldwide are affected by oral disorders, including gingivitis, caries, endodontic-periodontal diseases, and oral cancer, negatively impacting life quality and placing a massive financial burden on society. Besides affecting daily activities, poor oral health greatly increases several systemic disease risks, including diabetes and cardiovascular disease; therefore, developing more effective therapies to preserve oral and overall health is crucial.

Pathogenic cells, including bacteria, fungal, viral and cancer cells, are the leading causes of oral disorders. Accordingly, oral therapies aim to eliminate these cells instantly. For example, treatments for microbial infectious diseases rely primarily on antibiotic therapy. However, antimicrobial agents abuse has increased the concerns about bacterial drug resistance. Although surgery is the most frequent treatment for resectable tumors, incomplete or ineffective resection of lesions could result in local recurrence and a poor prognosis (1). Radiotherapy and chemotherapy can potentially result in serious systemic adverse effects and permanent damage to normal tissue due to toxicity (2).

**Figure 1:**
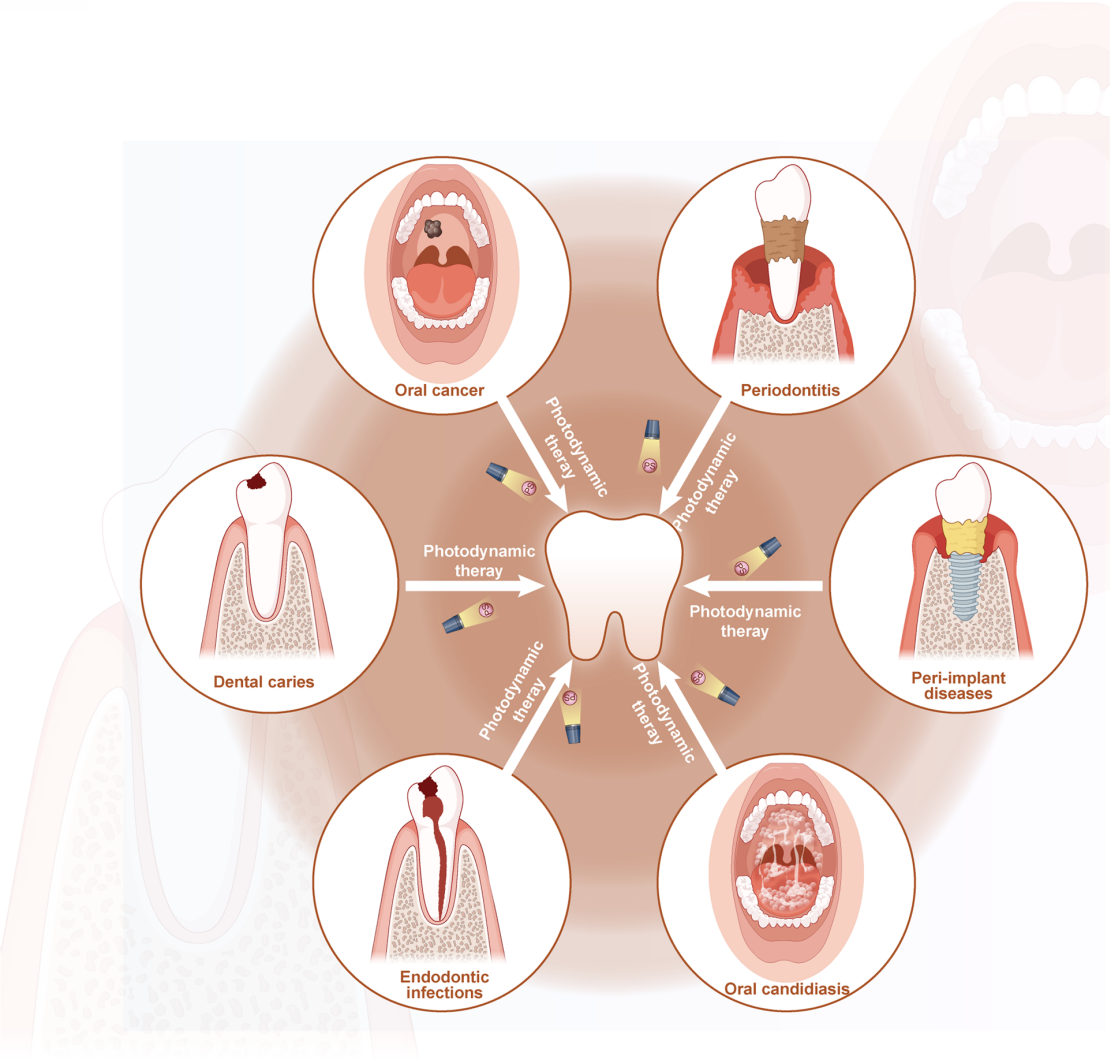
Schematic description of the application of PDT in treatment of oral diseases (by Figdraw).

Photodynamic therapy (PDT) is a non-invasive and secure therapeutic alternative for treating cancer and many other non-oncological diseases. Oscar Raab, who discovered that paramecia cultured with fluorescent dyes was killed upon exposure to light, first proposed the PDT idea in 1898 (3). The PDT has demonstrated beneficial application in various medical specialties, including dermatology, cancer, gynecology, and urology (4, 5). Using PDT in dentistry for conditions including oral cancer, potentially malignant disorders, periodontitis, dental caries, peri-implant disorders, and bacterial and fungal infections during root canal therapy is expanding rapidly (6, 7). The PDT offers better patient compliance than conventional surgery or drug therapy since it is non-invasive, safe, practical, and drug-resistant. Furthermore, PDT might be easily controlled by changing the parameters as needed to provide an accurate and personalized treatment. This review seeks to present a comprehensive overview of the applications of photodynamic therapy in the treatment of various oral diseases, including oral cancer, potentially malignant disorders, periodontitis, dental caries, peri-implant diseases, endodontic infections, and oral fungal infections (Figure 1).

## 2 Basic principles of photodynamic therapy

### 2.1 Mechanism of photodynamic therapy

**Figure 2:**
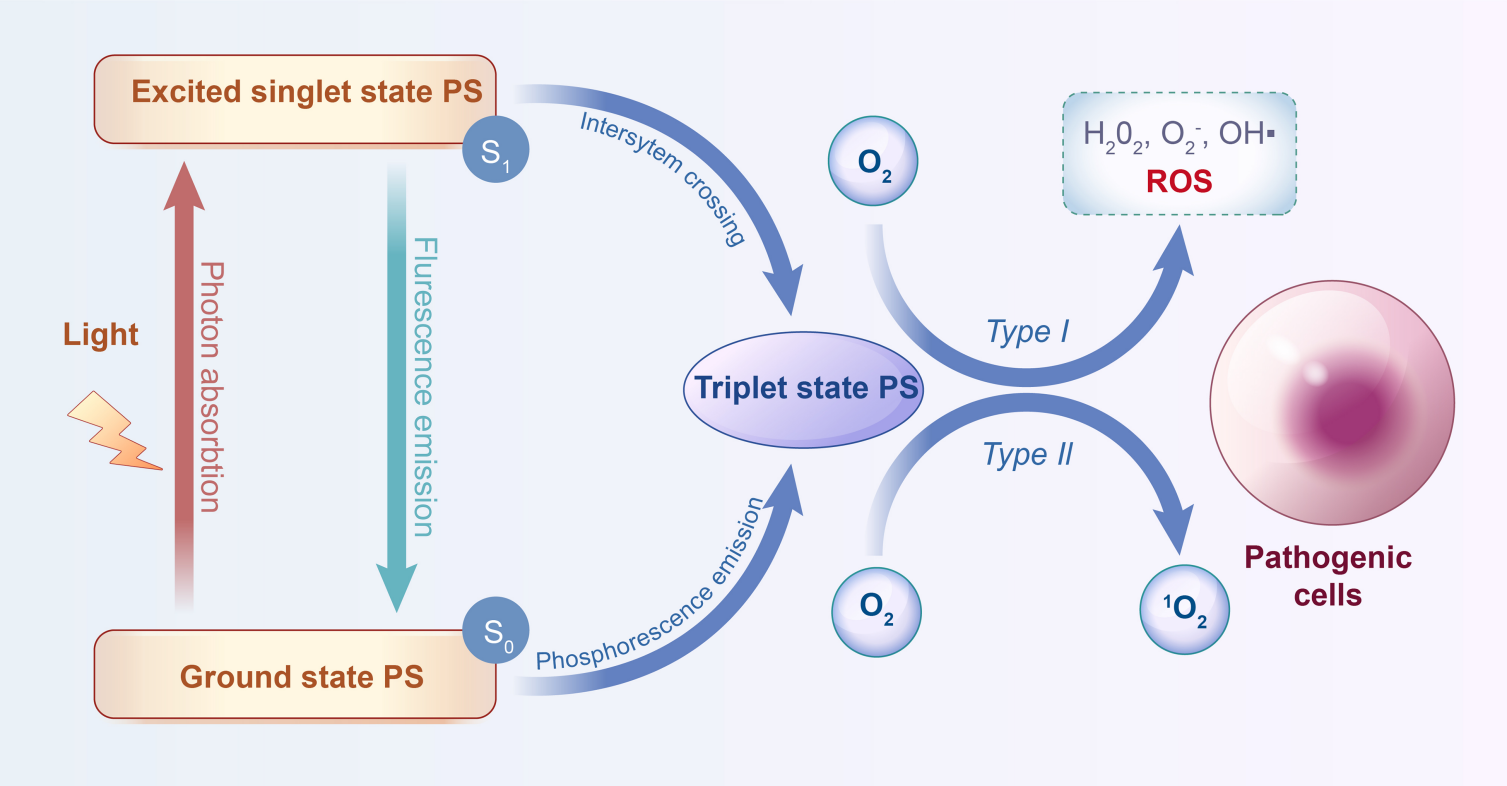
Schematic illustration of mechanism of the photodynamic reaction (by Figdraw).

PDT mechanism consists of three crucial elements: photosensitizer (PS), specified wavelength light source, and oxygen dissolved in the cells (8), which exclusively generates the desired outcomes through mutual interactions within pathological tissues. Figure 2 shows that the photodynamic reaction has two basic mechanisms, wholly dependent on oxygen molecules inside cells. Upon exposure to light, the photosensitizer transitions from a singlet basic energy level (S_0_) to an excited singlet state (S_1_). The excited singlet state could then transition to a triplet state (T_1_) or revert to the ground state with fluorescence emission. Reactive oxygen species (ROS) are created when the PS in the triplet state directly interacts with nearby biomolecules through hydrogen or electron transfer (Type I reaction); when this occurs, molecular oxygen is converted by the PS to highly reactive singlet oxygen (^1^O_2_) (Type II reaction) (4). Free radicals, including superoxide, hydroxyl, and lipid-derived radicals, are released due to type I reaction and attack cellular targets, resulting in direct cellular damage (9). Singlet excited-state oxygen is released in a type II reaction, which oxidizes lipids, proteins, and nucleic acids to cause cytotoxicity (10). Unsaturated lipids, comprising most cell and nuclear membranes, could react with the singlet oxygen. Consequently, these reaction byproducts may impede cell growth, trigger apoptosis in oral carcinoma cells, and significantly harm microorganisms.

### 2.2 Photosensitizers

PS development has progressed in recent decades of PDT research; a desirable PS should possess numerous important characteristics, including nontoxicity to surrounding tissues, hydrophilicity, high affinity and selectivity to target cells or microbes, and high quantum yield of photodynamic reaction (PDR). The first-generation typical photosensitizers, the hematoporphyrin derivative (HpD), and photofrin could be absorbed into the body for a long time but penetrate only into the tissue to a limited depth (<0.5 cm) (11). Porfifimer sodium, marketed under photofrin, was authorized for treating Barrett's esophagus in 2003 and early-stage lung cancer in 1998. The absorbance spectrum, tissue selectivity, and ROS production efficiency of the second-generation PSs have been enhanced. A naturally occurring pro-photosensitizer and a precursor to hemoglobin manufacture is 5-aminolaevulinic acid (ALA). A surplus of ALA leads tumor cells to absorb ALA rapidly but slowly degrade protoporphyrin IX (PpIX), causing PpIX photosensitizer to accumulate. Numerous second-generation photosensitizers are available, including phthalocyanines, verteporfin (VP), meta-tetrahydroxyphenylchlorin (m-THPC), and palladium bacteriopheophorbide. A light bleaching capability, antibody conjugates, or a protein/receptor system were added to photosensitizers in the third generation, improving the ability of a photosensitizer to target tumor tissues more effectively. The antibody affinity for tumor cells increases when conjugates to photosensitizers, decreasing healthy cell location. Furtherer more, toluidine blue (TB), methylene blue (MB), and erythrosine have been widely used for antimicrobial photodynamic therapy (12, 13). Besides photosensitizers, certain inorganic salts (such as potassium iodide and potassium bromide) the inhibition or deterioration of target cells and effectively inhibited or eradicated bacterial and fungal biofilms (14–16).

The selection of an appropriate photosensitizer for PDT is a crucial decision influenced by several factors, including the specific type of oral disease being addressed (whether antitumor or antimicrobial), the chemical properties of the photosensitizer, its capacity to generate reactive oxygen species, as well as its toxicity, selectivity, and availability. In addition to these intrinsic properties, the formulation and delivery method of the photosensitizer can significantly impact its therapeutic performance. Utilizing a molecular carrier, including a liposome nano species, is an alternative strategy. To maintain a high concentration in the targeted tissues, these modified photosensitizers with poor solubility in aqueous solutions are prevented from being delivered into the bloodstream.

### 2.3 Light source

Lamps, light-emitting diodes (LEDs), and lasers are the main light sources in PDT that are chosen based on the target tissue location, photosensitizer type, and administration dose. Lasers produce a coherent, focused, monochromatic light intensity, widely used to perform superficial and interstitial PDT; its limited clinical applicability is the monochromatic nature and high cost. The LED is becoming increasingly popular for PDT as a viable alternative to lasers because of their low cost, easy manipulation, and access to tissue surfaces. The LED semiconductor device uses electron-hole recombination to produce light: large beam divergence and wide spectral breadth enable LEDs to excite numerous PSs in their emission spectrum simultaneously. Lamps were the first artificial light sources utilized in PDT studies (17). Theoretically, lamps could couple to light guides to concentrate the light on particular therapy lesions; however, the coupling losses are substantial. Consequently, lamps are better suited to treating superficial malignancies such as skin or oral cavities. Compared with lasers or LEDs, the lamps (300–1,200 nm) with appropriate optical filtering could match any photosensitizer, but they have poor monochromaticity, insufficient intensity, and low energy.

The properties of the light source, whether it is a laser or a LED, play a pivotal role in the effectiveness of PDT. Primarily, each photosensitizer requires activation by light at a wavelength that aligns with its specific absorption characteristics. For example, certain photosensitizers, such as mTHPC, porfimer sodium, and ALA, are activated by red light, which typically spans a wavelength range of 600–700 nm. This wavelength range is advantageous for deeper tissue penetration, rendering it suitable for the treatment of oral cavity cancers. Another critical attribute is the intensity of the light source. The light must possess sufficient intensity to activate the photosensitizer while avoiding thermal damage to adjacent tissues. Low-power light sources are often favored as they reduce the risk of heat-induced damage while still effectively activating the photosensitizer. Additionally, the coherence and collimation of the light are important considerations. Lasers, which emit coherent and collimated light, are frequently employed in PDT due to their ability to deliver light precisely to the target area, thereby enabling controlled activation of the photosensitizer. LEDs, which emit non-coherent light, are increasingly utilized due to their cost-effectiveness and ease of use. LEDs are advantageous for treating larger surface areas as they provide a broader area of illumination. Critical parameters in this context include the duration of exposure and the total energy delivered, known as fluence. The light source must deliver an adequate dose of energy to effectively activate the photosensitizer. The optimal fluence is contingent upon the specific application and the photosensitizer employed. In conclusion, the ideal light source for PDT should possess a wavelength that aligns with the absorption spectrum of the photosensitizer, sufficient intensity to activate the photosensitizer without inducing thermal damage, and the capability to deliver the appropriate fluence to the target tissue.

## 3 Oral cavity cancers and potentially malignant disorders

Oral cavity cancers are prevalent globally, accounting for nearly 202,000 new cases yearly (18). Additionally, oral squamous cell carcinoma (OSCC) accounts for nearly 90% of oral cavity malignancies, with a global incidence anticipated at 377,713 new cases and 177,757 deaths in 2020 (19). Oral cancer oncogenesis is influenced by several variables and developmental stages. The malignant development of oral potentially malignant disorders (OPMDs) including actinic cheilitis (AC), oral leukoplakia (OL), oral lichen planus (OLP), oral erythroleukoplakia (OEL), oral erythroplakia (OE), oral submucosal fibrosis, proliferative verrucous leukoplakia (PVL), and epithelial dysplasia can be viewed through oral carcinogenesis studies. Recently, it has become widely accepted. Patients with OPMDs have a 5–100 fold higher risk of developing malignant transformation than the general population (20); OL, OE, and PVL exhibited a 15%–90% malignant transformation rate (21). The most popular forms of oral cancer treatment include surgery, chemotherapy, and radiation therapy, all of which have clear drawbacks. Oral cancer surgery could alter the facial appearance, and radiation therapy, chemotherapy, and surgery all have severe functional side effects that can affect the ability of a patient to chew, speak, swallow, and taste (22). PDT may be appropriate for patients with significant lesions in areas with high cosmetic value or who refuse standard invasive surgery.

Hopper et al. (23) conducted a study that was open-label and multicenter in nature. The purpose of the study was to investigate the effectiveness of administering 0.15 mg/kg of mTHPC through intravenous injection in conjunction with a 20 J/cm^2^ red laser (652 nm) for the treatment of early-stage OSCC at stages Tis, T1, or T2, N0M0. The results indicated that 85% of the patients who followed the treatment (97 out of 114) experienced full tumor responses. The response rate remained constant at 85% (95% CI: 77%–93%) after one year and 77% (95% CI: 66%–87%) after two years. The actuarial survival rates at one and two years were 89% (95% CI: 83%–95%) and 75% (95% CI: 66%–84%), respectively. Researchers also recorded remarkable aesthetic and functional outcomes, with no significant adverse effects on tissue integrity, speech, or swallowing ability.

A study conducted by Han et al. showcased the therapeutic effectiveness of ALA-PDT in the treatment of oral leukoplakia in Chinese patients (24). The therapy consisted of applying a 20% ALA gel and exposing the area to a 632 nm laser with an intensity of 500 mW/cm^2^ and a 90–180 J/cm^2^ dose. The overall response rate was 86.2%, with 55.2% of participants achieving complete remission. In a separate investigation, Yao et al. (25) conducted a comparison between the impacts of ablative fractional laser-assisted photodynamic treatment (AFL-PDT) and ablative fractional laser (AFL) alone for oral leukoplakia. The findings revealed a notably greater percentage of successful treatment in the AFL-PDT group (100%) than in the AFL group (80.9%), with a difference of 19.1% (95% CI: 0.7%–40.0%). In addition, the AFL-PDT group demonstrated decreased recurrence rates at 6 and 12 months after therapy. Neither group experienced any serious adverse events or systemic effects.

Schuch et al. (26) extensively examined the effects of PDT on treating OPMD and OSCC. Fourteen different types of photosensitizing agents (ALA, chlorine-e6, foscan, and so on) with a light source irradiation at 417–670 nm, 10–500 mW/cm^2^, 1.5–200 J/cm^2^, and 0.5–143 min was used. The analysis included 9,245 people with OPMD (*n* = 7,487) or OSCC (*n* = 1,758). OEL (93%; 100 cases) and AC (67.6%; 448 cases) had the highest complete response rates among the informed cases; conversely, OLP had a 42.9% complete response rate. Eight studies were conducted to investigate the OL, OE, OEL, or both, revealing that OL responded less strongly than OE and OEL. Less than half of OL cases receive a complete answer, compared with over 90% of instances for OE and OEL. Four investigations (*n* = 411) on OSCC demonstrated complete resolution in 83.4% of the cases and nonresolution in 16.6% of instances; a follow-up of 28.4 months showed a 12% recurrence rate.

PDT is commonly used to treat OPMD or early-stage oral cavity cancers without nodal metastases (Cis, T1, T2), either as the main treatment or as an alternate option (Tables 1, 2). The greatest positive reactions are seen in surface tumors that fall within the light source's permeability region, which is between 5 and 10 mm (27). Although PDT provides favorable cosmetic results and preserves functionality with low invasiveness and no toxicity (28), it is crucial to acknowledge that its efficacy is restricted to treating superficial early-stage lesions and does not affect lymph nodes. Moreover, PDT can serve as an adjunctive therapy for treating remaining margins following surgical procedures or in conjunction with other therapies.

**Table 1:** Summary of studies on PDT for the treatment of oral cavity cancers.

	Study	Year	Study design	Treatment groups	Lesion type	Clinical inclusion standard	Type of photosensitizers	Photosensitizers dose	Light source	Irradiation mode	Wave length	Fluence rate/Power	Energy density	Irradiation time	Treatment sessions	Treatment interval	Follow-up	Primary outcome	Referrence
1	Kübler et al.	2001	Prospective, open label, multi-centre study	–	Primary SCC of the lip (Tis; T1; T2)	Tis; T1; T2 tumour (≤2.5 cm diameter, ≤0.5 cm depth)	Foscan (mTHPC)	0.15 mg/kg	Argon-dye laser system or diode laser	–	652 nm	100 mW/cm^2^	20 J/cm^2^	–	–	–	12 weeks (clinical and ultrasonic examination); 424 days (mean follow up)	PDT offers an interesting alternative for the treatment of SCC of the lip.	Kübler, A. C., de Carpentier, J., Hopper, C., Leonard, A. G., & Putnam, G. (2001). Treatment of squamous cell carcinoma of the lip using Foscan-mediated photodynamic therapy. International journal of oral and maxillofacial surgery, 30(6), 504–509. doi: 10.1054/ijom.2001.0160
2	Copper et al.	2003	Prospective study	–	SCC of the oral cavity or pharynx	Stage I: 68% stage II: 28% multiple primary tumors: 8%	mTHPC	0.15 mg/kg	Diode laser	–	652 nm	100 mW/cm^2^	20 J/cm^2^	–	–	–	Every month (the first year), every 2 months(the second year) and every 3 months (the third year); 37 months (mean follow up)	PDT could be considered as an alternative to surgery or radiotherapy in specific cases of head and neck cancer.	Copper, M. P., Tan, I. B., Oppelaar, H., Ruevekamp, M. C., & Stewart, F. A. (2003). Meta-tetra(hydroxyphenyl)chlorin photodynamic therapy in early-stage squamous cell carcinoma of the head and neck. Archives of otolaryngology–head & neck surgery, 129(7), 709–711. doi: 10.1001/archotol.129.7.709
3	Hopper et al.	2004	Multicentre, open-label, single-group Phase IIb study	–	Primary SCC of the lip, oral cavity, oropharynx/hypopharynx	Tis, T1 or T2, N0, M0 tumour (≤2.5 cm diameter, ≤0.5 cm estimated depth)	mTHPC	0.15 mg/kg	Laser	–	652 nm	100 mW/cm^2^	20 J/cm^2^	200 s	–	–	12 weeks (biopsy); followed up for 2 years after PDT	mTHPC-mediated PDT is an effective method of dealing with early oral squamous cell carcinoma.	Hopper, C., Kübler, A., Lewis, H., Tan, I. B., & Putnam, G. (2004). mTHPC-mediated photodynamic therapy for early oral squamous cell carcinoma. International journal of cancer, 111(1), 138–146. doi: 10.1002/ijc.20209
4	Copper et al.	2007	Retrospective study	–	SCC of the oral cavity or oropharynx	Infiltrate <5 mm	mTHPC	0.15 mg/kg	Diomed laser or Ceralas laser	–	652 nm	100 mW/cm^2^	20 J/cm^2^	15–30 min	1–2	–	Every month (the first year), every 2 months (the second year) and hereafter once every 3 months	PDT is a convenient and effective treatment for the early-stage new primary head and neck tumours.	Copper, M. P., Triesscheijn, M., Tan, I. B., Ruevekamp, M. C., & Stewart, F. A. (2007). Photodynamic therapy in the treatment of multiple primary tumours in the head and neck, located to the oral cavity and oropharynx. Clinical otolaryngology: official journal of ENT-UK; official journal of Netherlands Society for Oto-Rhino-Laryngology & Cervico-Facial Surgery, 32(3), 185–189. doi: 10.1111/j.1365-2273.2007.01441.x
5	Jerjes et al.	2011	Prospective cohort clinical study	–	Oral SCC	T1N0: 9 patients T2N0: 29 patients	mTHPC	0.15 mg/kg	Diode laser	–	652 nm	100 mW/cm^2^	10–20 J/cm^2^	200 s	1–3	–	4 weeks (first follow-up); ≥5 years	mTHPC-PDT is a comparable modality with reduced morbidity in managing low-risk (T1/T2 N0) tumors of the oral cavity.	Jerjes, W., Upile, T., Hamdoon, Z., Alexander Mosse, C., Morcos, M., & Hopper, C. (2011). Photodynamic therapy outcome for T1/T2 N0 oral squamous cell carcinoma. Lasers in surgery and medicine, 43(6), 463–469. doi: 10.1002/lsm.21071
6	Jerjes et al.	2011	Preliminary study	–	Advanced and/or recurrent tongue base carcinoma	Stage IV	mTHPC	0.15 mg/kg	Diode laser	–	652 nm	–	20 J/cm^2^	–	–	–	Approximately 6 weeks (clinical assessment); 48 months (mean)	Two-thirds of the patients responded well to the treatment, and a third had a moderate response. PDT is a successful modality for treating advanced and/or recurrent tongue base carcinoma.	Jerjes, W., Upile, T., Radhi, H., & Hopper, C. (2011). Photodynamic therapy and end-stage tongue base cancer: short communication. Head & neck oncology, 3, 49. doi: 10.1186/1758-3284-3-49
7	Jerjes et al.	2011	Prospective study	–	Tongue base squamous cell and Adenoid cystic carcinoma	Stage IV	mTHPC	0.15 mg/kg	Diode laser	–	652 nm	100 mW/cm^2^	20 J/cm^2^	200 s	1–3	–	Approximately 6 weeks (clinical assessment); 36 ± 8.8 months (mean)	More than 50% patients showed “good response” to the treatment and about a third reported ‘‘moderate response”. PDT is a successful palliative approach for treating advanced and/or recurrent tongue base carcinoma.	Jerjes, W., Upile, T., Hamdoon, Z., Abbas, S., Akram, S., Mosse, C. A., Morley, S., & Hopper, C. (2011). Photodynamic therapy: The minimally invasive surgical intervention for advanced and/or recurrent tongue base carcinoma. Lasers in surgery and medicine, 43(4), 283–292. doi: 10.1002/lsm.21048
8	Karakullukcu et al.	2011	Retrospective study	–	Oral cavity and oropharynx SCC or CIS	Early stage (Tis, T1, T2) and ≤5 mm depth	mTHPC	0.15 mg/kg	Diode laser	–	652 nm	–	20 J/cm^2^	–	–	–	1, 2, 4, 8, 16, 24, 36, 52 weeks	The overall response rate is 90.7% with a complete response rate of 70.8%.	Karakullukcu, B., van Oudenaarde, K., Copper, M. P., Klop, W. M., van Veen, R., Wildeman, M., & Bing Tan, I. (2011). Photodynamic therapy of early stage oral cavity and oropharynx neoplasms: an outcome analysis of 170 patients. European archives of oto-rhino-laryngology: official journal of the European Federation of Oto-Rhino-Laryngological Societies (EUFOS): affiliated with the German Society for Oto-Rhino-Laryngology—Head and Neck Surgery, 268(2), 281–288. doi: 10.1007/s00405-010-1361-5
9	Karakullukcu et al.	2012	Prospective study	–	Nonmetastatic recurrent SCCs (base of tongue)	After (chemo)radiotherapy	mTHPC	0.15 mg/kg	Diode laser	–	652 nm	–	30 J/cm^2^	300 s	–	–	Every 2 weeks (the first 2 months) and thereafter every month until death	PDT can be a curative alternative to palliative care to nonmetastatic recurrent base of tongue tumors when conventional curative techniques are exhausted.	Karakullukcu, B., Nyst, H. J., van Veen, R. L., Hoebers, F. J., Hamming-Vrieze, O., Witjes, M. J., de Visscher, S. A., Burlage, F. R., Levendag, P. C., Sterenborg, H. J., & Tan, I. B. (2012). mTHPC mediated interstitial photodynamic therapy of recurrent nonmetastatic base of tongue cancers: Development of a new method. Head & neck, 34(11), 1597–1606. doi: 10.1002/hed.21969
10	Karakullukcu et al.	2013	Comparative Study	Group 1: Surgery group Group 2: PDT group	Primary oral SCC	T1/T2 and ≤5 mm depth	mTHPC	–	–	–	–	–	–	–	–	–	≥2 years	PDT is comparable to trans-oral resection of early stage SCC of the oral cavity regarding survival and disease control.	Karakullukcu, B., Stoker, S. D., Wildeman, A. P., Copper, M. P., Wildeman, M. A., & Tan, I. B. (2013). A matched cohort comparison of mTHPC-mediated photodynamic therapy and trans-oral surgery of early stage oral cavity squamous cell cancer. European archives of oto-rhino-laryngology: official journal of the European Federation of Oto-Rhino-Laryngological Societies (EUFOS): affiliated with the German Society for Oto-Rhino-Laryngology—Head and Neck Surgery, 270(3), 1093–1097. doi: 10.1007/s00405-012-2104-6
11	Durbec et al.	2013	Retrospective study	–	Recurrent oral cavity or oropharyngeal carcinoma or second appearance of tumor in previously irradiated	tumor thickness ≤1 cm	mTHPC	0.15 mg/kg	Diode laser	–	652 nm	100 mW/cm^2^	20 J/cm^2^	–	–	–	Weekly monitoring (the first month), 2-monthly (the first year), 3-monthly (second and third years), 4-monthly (the fourth and fifth years), then every 2 years up to death	PDT provides useful results in terms of survival and improvement in quality of life treatment of recurrent carcinoma of the oral cavity and oropharynx.	Durbec, M., Cosmidis, A., Fuchsmann, C., Ramade, A., & Céruse, P. (2013). Efficacy and safety of photodynamic therapy with temoporfin in curative treatment of recurrent carcinoma of the oral cavity and oropharynx. European archives of oto-rhino-laryngology: official journal of the European Federation of Oto-Rhino-Laryngological Societies (EUFOS): affiliated with the German Society for Oto-Rhino-Laryngology—Head and Neck Surgery, 270(4), 1433–1439. doi: 10.1007/s00405-012-2083-7
12	de Visscher et al.	2013	Retrospective study	Group 1: Surgery group Group 2: PDT group	Primary OSCC	Early stage (cT1–2N0) and ≤5 mm depth	mTHPC	0.15 mg/kg	Diode laser	–	652 nm	100 mW/cm^2^	20 J/cm^2^	–	–	–	Surgery group: 67 months (mean) PDT group: 33 months (mean)	In the treatment of T1 tumors, the efficacy of PDT is similar to surgery. In the treatment of T2 tumors, surgery is more effective. PDT and surgery showed similar overall survival rates for both T1 and T2 tumors.	de Visscher, S. A., Melchers, L. J., Dijkstra, P. U., Karakullukcu, B., Tan, I. B., Hopper, C., Roodenburg, J. L., & Witjes, M. J. (2013). mTHPC-mediated photodynamic therapy of early stage oral squamous cell carcinoma: a comparison to surgical treatment. Annals of surgical oncology, 20(9), 3076–3082. doi: 10.1245/s10434-013-3006-6
13	Toratani et al.	2015	Retrospective study	–	Superficial oral SCC or CIS	≤5 mm depth	mTHPC	2 mg/kg	Excimer dye laser	Pulse frequency: 40 Hz	630 nm	160 mW	100–150 J/cm^2^	–	–	–	Approximately once a month in the year after treatment and every three months subsequently	A comparable modality with reduced morbidity in managing low-risk tumors.	Toratani, S., Tani, R., Kanda, T., Koizumi, K., Yoshioka, Y., & Okamoto, T. (2016). Photodynamic therapy using Photofrin and excimer dye laser treatment for superficial oral squamous cell carcinomas with long-term follow up. Photodiagnosis and photodynamic therapy, 14, 104–110. doi: 10.1016/j.pdpdt.2015.12.009
14	Rigual et al.	2009	Prospective clinical trial	–	Dysplasia	Moderate to severe	Porfimer sodium	2 mg/kg	Argon-pumped dye laser or diode laser	–	630 nm	–	50 J/cm^2^	–	–	–	1 week, 1 month and 3-month intervals thereafter	PDT with porfimer sodium is an effective alternative treatment in dysplasias, CIS, and early carcinomas of the larynx and the oral cavity. Compared to T1 carcinomas, oral cavity dysplasias showed a weaker response.	Rigual, N. R., Thankappan, K., Cooper, M., Sullivan, M. A., Dougherty, T., Popat, S. R., Loree, T. R., Biel, M. A., & Henderson, B. (2009). Photodynamic therapy for head and neck dysplasia and cancer. Archives of otolaryngology–head & neck surgery, 135(8), 784–788. doi: 10.1001/archoto.2009.98
					Squamous CIS of the oral cavity or larynx	Stage I (T1N0) and ≤3 mm depth							75 J/cm^2^						
15	Narahara et al.	2023	Cohort clinical studyy	–	OSCC or Epithelial dysplasia	T1N0M0 and ≤5 mm depth (for OSCC)	Porfimer sodium	2 mg/kg	Excimer dye laser	Irradiation output: 4 mJ/pulse/cm^2^ repetition rate: 40 Hz	630 nm	–	100 J/cm^2^	–	–	–	106 ± 43.4 months	PDT offers dependable short-term results for OSCC and ED, though its long-term effects are limited.	Narahara, S., Ikeda, H., Ogata, K., Shido, R., Asahina, I., & Ohba, S. (2023). Long-term effect of photodynamic therapy on oral squamous cell carcinoma and epithelial dysplasia. Photodiagnosis and photodynamic therapy, 41, 103246. doi: 10.1016/j.pdpdt.2022.103246
							Talaporfin sodium	40 mg/kg	Semiconductor laser	–	664 nm	–	100 J/cm^2^	–	–	–			
16	Ikeda et al.	2018	Prospective study	–	Oal SCC	No metastasis and ≤10 mm depth	Talaporfin sodium	40 mg/m^2^	Semiconductor laser	Continuous wave	664 nm	500 mW (maximum)	100 J/cm^2^	–	–	–	≥4 years	Out of eight cases, six achieved complete response (CR), while two exhibited a partial response (PR) as a clinical outcome of t-PDT. t-PDT is an effective modality for oral SCC.	Ikeda, H., Ohba, S., Egashira, K., & Asahina, I. (2018). The effect of photodynamic therapy with talaporfin sodium, a second-generation photosensitizer, on oral squamous cell carcinoma: A series of eight cases. Photodiagnosis and photodynamic therapy, 21, 176–180. doi: 10.1016/j.pdpdt.2017.11.016
17	Ikeda et al.	2013	Retrospective study	–	SCC in the oral cavity	T1N0M0 to T2N0M0 (≤4 cm diameter, ≤5 mm depth)	Photofrin(®)	2 mg/kg	Excimer dye laser	Irradiation output: 4 mJ/pulse/cm^2^ repetition rate: 40 Hz	630 nm	–	100 J/cm^2^	30–150 min	1–2	–	1, 2, 4, 8, 16, 24, 36, 52 weeks and 2 years	A complete response was observed in 94.4% of patients (17 out of 18) in the SCC group and in 100% of patients (7 out of 7) in the epithelial dysplasia group. Overall, the disease specific survival rate was 95.8%.	Ikeda, H., Tobita, T., Ohba, S., Uehara, M., & Asahina, I. (2013). Treatment outcome of Photofrin-based photodynamic therapy for T1 and T2 oral squamous cell carcinoma and dysplasia. Photodiagnosis and photodynamic therapy, 10(3), 229–235. doi: 10.1016/j.pdpdt.2013.01.006
					Oral mucosal dysplasia	Moderate to severe													
18	Wenig et al.	1990	Clinical Trial	–	SCC of the head and neck	Early stage and ≤10 mm depth	Photofrin II	2 mg/kg	R argon-pumped dye laser	–	630 nm	140 mW/cm^2^ (maximum)	75 J/cm^2^	–	–	–	6–51 months	PDT can provoke a significant clinical and histological reaction in most head and neck cancers that are treated.	Wenig, B. L., Kurtzman, D. M., Grossweiner, L. I., Mafee, M. F., Harris, D. M., Lobraico, R. V., Prycz, R. A., & Appelbaum, E. L. (1990). Photodynamic therapy in the treatment of squamous cell carcinoma of the head and neck. Archives of otolaryngology–head & neck surgery, 116(11), 1267–1270. doi: 10.1001/archotol.1990.01870110039003
19	Rigual et al.	2013	Phase I, open label study	–	Dysplasia	Moderate to severe	HPPH	4 mg/m^2^	Tunable dye laser	–	665 nm	–	50–140 J/cm^2^	–	–	–	1 week, 1 month, 3 months and 3 to 6 months intervals thereafter; range from 5 to 40 months	HPPH-PDT is safe for the treatment of carcinoma in situ/dysplasia and early-stage cancer of the oral cavity.	Rigual, N., Shafirstein, G., Cooper, M. T., Baumann, H., Bellnier, D. A., Sunar, U., Tracy, E. C., Rohrbach, D. J., Wilding, G., Tan, W., Sullivan, M., Merzianu, M., & Henderson, B. W. (2013). Photodynamic therapy with 3-(1'-hexyloxyethyl) pyropheophorbide a for cancer of the oral cavity. Clinical cancer research: an official journal of the American Association for Cancer Research, 19(23), 6605–6613. doi: 10.1158/1078-0432.CCR-13-1735
					SCC of the oral cavity	situ or T1 and ≤4 mm depth													
20	Wang et al.	2021	Retrospective clinical study	–	Cancerous lesions of the gingiva and oral mucosa	Single neoplastic lesion	ALA	10%	LED	–	635 nm	100 mW/cm^2^	100 J/cm^2^	–	4	14 days	Median: 34.1 months (range: 26–43 months)	ALA-PDT is a safe and beneficial addition to ICT for patients with localized OSCC.	Wang, X., Li, N., Meng, J., & Wen, N. (2021). The use of topical ALA-photodynamic therapy combined with induction chemotherapy for locally advanced oral squamous cell carcinoma. American journal of otolaryngology, 42(6), 103112. doi: 10.1016/j.amjoto.2021.103112
21	Siddiqui et al.	2022	Clinical validation study	–	Buccal mucosal cancer	T1N0M0 and ≤5 mm depth, 20 mm width	ALA	60 mg/kg, divided doses	LED	3–5 fractions	635 nm	33–54 mW/cm^2^	100 J/cm^2^	30–50 min	–	–	54 weeks (range 14–141, median 50)	ALA-PDT has considerable potential to reduce the morbidity and mortality associated with oral cancer.	Siddiqui, S. A., Siddiqui, S., Hussain, M. A. B., Khan, S., Liu, H., Akhtar, K., Hasan, S. A., Ahmed, I., Mallidi, S., Khan, A. P., Cuckov, F., Hopper, C., Bown, S., Celli, J. P., & Hasan, T. (2022). Clinical evaluation of a mobile, low-cost system for fluorescence guided photodynamic therapy of early oral cancer in India. Photodiagnosis and photodynamic therapy, 38, 102843. doi: 10.1016/j.pdpdt.2022.102843

ALA, 5-aminolevulinic acid; cm^2^, square centimeter; CIS, carcinoma in situ; HPPH, 3-(1′-hexyloxyethyl) pyropheophorbide; ICT, induction chemotherapy; m, minutes; mTHPC, meso-tetrahydroxyphenyl chlorin (meta-tetrahydroxy-phenyl chlorin); nm, nanometers; mW, milliwatts; s, seconds; SCC, squamous cell carcinoma;-, not reported.

**Table 2:** Summary of studies on PDT for the treatment of potentially malignant disorders.

	Study	Year	Study design	Treatment groups	Lesion type	Type of photosensitizers	Photosensitizers dose	Light source	Irradiation mode	Wave length	Fluence rate/Power	Energy density	Irradiation time	Treatment sessions	Treatment interval	Follow-up	Primary outcome	Referrence
1	Kübler et al.	1998	Before and after clinical study	–	OL	ALA	20%	Argon-pumped dye laser	–	630 nm	100 mW/cm^2^	100 J/cm^2^	1,000 s	1	–	6–16 (9, mean) months	The use of ALA-PDT might be a promising option for treating oral leukoplakia.	Kübler, A., Haase, T., Rheinwald, M., Barth, T., & Mühling, J. (1998). Treatment of oral leukoplakia by topical application of 5-aminolevulinic acid. International journal of oral and maxillofacial surgery, 27(6), 466–469. doi: 10.1016/s0901-5027(98)80040-4
2	Sieron et al.	2003	Single arm clinical trial	–	OL	ALA	10%	Argon-pumped dye laser	–	635 nm	150 mW/cm^2^	100 J/cm^2^	–	6–8	–	4 to 34 months	CR-10, NR-2; 1 recurrence after 6 months. PDT seems to be a viable option compared to traditional OL treatments.	Sieroń, A., Adamek, M., Kawczyk-Krupka, A., Mazur, S., & Ilewicz, L. (2003). Photodynamic therapy (PDT) using topically applied delta-aminolevulinic acid (ALA) for the treatment of oral leukoplakia. Journal of oral pathology & medicine: official publication of the International Association of Oral Pathologists and the American Academy of Oral Pathology, 32(6), 330–336. doi: 10.1034/j.1600-0714.2003.00068.x
3	Chen et al.	2005	Clinical trial	–	OL and OVH	ALA	5%	LED	Five 3 min and one 100 s irradiations separated by five 3 min rests	635 ± 5 nm	100 mW/cm^2^	100 J/cm^2^	1,000 s	1–3	0.5 week	OVH: 5–14 months OL: 3–12 months	Complete regression of OVH lesions can be accomplished with fewer than six weekly treatments of ALA-PDT. OL lesions that receive treatment twice weekly show significantly better clinical outcomes compared to those treated once weekly.	Chen, H. M., Yu, C. H., Tu, P. C., Yeh, C. Y., Tsai, T., & Chiang, C. P. (2005). Successful treatment of oral verrucous hyperplasia and oral leukoplakia with topical 5-aminolevulinic acid-mediated photodynamic therapy. Lasers in surgery and medicine, 37(2), 114–122. doi: 10.1002/lsm.20214
4	Chen et al.	2007	Interventional before and after study	–	OL, OEL and OVH	ALA	20%	LED	Five 3-min and one 100 s irradiations	635 ± 5 nm	100 mW/cm^2^	100 J/cm^2^	1,000 s	≤8	0.5 or 1 week	Once a month for 3–42 months	Complete regression of OVH lesions can be achieved by less than seven treatments of ALA-PDT once a week. OL lesions treated twice a week have a significantly better clinical outcome than OL lesions treated once a week. OEL lesions treated once a week have a significantly better clinical outcome than OL lesions treated once a week.	Chen, H. M., Yu, C. H., Tsai, T., Hsu, Y. C., Kuo, R. C., & Chiang, C. P. (2007). Topical 5-aminolevulinic acid-mediated photodynamic therapy for oral verrucous hyperplasia, oral leukoplakia and oral erythroleukoplakia. Photodiagnosis and photodynamic therapy, 4(1), 44–52. doi: 10.1016/j.pdpdt.2006.11.003
5	Shafirstein et al.	2011	Single arm, single-site phase 1/2 pilot study	–	OL	ALA	20%	Pulsed dye laser	1.5 ms pulses, 1–3 s intervals and 7 mm-diameter spot size	585 nm	–	6–8 J/cm^2^	–	1–2	–	30, 90, and 365 days	Using PDT with 5-aminolevulinic acid alongside a pulsed dye laser could lead to the regression of oral leukoplakia.	Shafirstein, G., Friedman, A., Siegel, E., Moreno, M., Bäumler, W., Fan, C. Y., Morehead, K., Vural, E., Stack, B. C., Jr, & Suen, J. Y. (2011). Using 5-aminolevulinic acid and pulsed dye laser for photodynamic treatment of oral leukoplakia. Archives of otolaryngology–head & neck surgery, 137(11), 1117–1123. doi: 10.1001/archoto.2011.178
6	Kawczyk-Krupka et al.	2012	Comparative Study	Group 1: PDT Group 2: Cryosurgery	OL	ALA	20%	Diomed 630 laser	–	630 nm	–	100 J/cm^2^	15 min	2–11	–	4–34 months	In PDT group, a complete response was obtained in 35 patients (72.9%), with thirteen recurrences observed (27.1%) over a six-month period. PDT appears to be a treatment option that might act as an alternative to traditional surgery for oral leukoplakia.	Kawczyk-Krupka, A., Waśkowska, J., Raczkowska-Siostrzonek, A., Kościarz-Grzesiok, A., Kwiatek, S., Straszak, D., Latos, W., Koszowski, R., & Sieroń, A. (2012). Comparison of cryotherapy and photodynamic therapy in treatment of oral leukoplakia. Photodiagnosis and photodynamic therapy, 9(2), 148–155. doi: 10.1016/j.pdpdt.2011.12.007
							10%	Argon-pumped dye laser	–	635 nm	–	100 J/cm^2^	15 min	3–12	–			
7	Wong et al.	2013	Clinical trial	–	OL	ALA	30, 60 mg/kg	Long pulse dye laser	–	585 nm	100 mW/cm^2^	2, 4 J/cm^2^	–	–	–	2 years	ALA PDT was safely given with a light dose of up to 4 J/cm^2^ and showed effectiveness.	Wong, S. J., Campbell, B., Massey, B., Lynch, D. P., Cohen, E. E. W., Blair, E., Selle, R., Shklovskaya, J., Jovanovic, B. D., Skripkauskas, S., Dew, A., Kulesza, P., Parimi, V., Bergan, R. C., & Szabo, E. (2013). A phase I trial of aminolevulinic acid-photodynamic therapy for treatment of oral leukoplakia. Oral oncology, 49(9), 970–976. doi: 10.1016/j.oraloncology.2013.05.011
8	Selvam et al.	2015	Pilot study	–	OL	ALA	10%	Xenon lamp	–	630 ± 5 nm	100 mW/cm^2^	100 J/cm^2^	1,000 s	6–8	1 week	1 year	Complete response: Two patients; partial response: Two patients; and no response: One patient. ALA-PDT appears to be a promising option for treating OL.	Selvam, N. P., Sadaksharam, J., Singaravelu, G., & Ramu, R. (2015). Treatment of oral leukoplakia with photodynamic therapy: A pilot study. Journal of cancer research and therapeutics, 11(2), 464–467. doi: 10.4103/0973-1482.147703
9	Maloth et al.	2016	Clinical trial	Control group: conventional therapy Study group: PDT	OL and OLP	ALA	5%	LED	–	420 nm	>500 mW/cm^2^	–	10 min	1	–	No follow up	PDT seems to be a viable option compared to traditional treatments for oral premalignant lesions.	Maloth, K. N., Velpula, N., Kodangal, S., Sangmesh, M., Vellamchetla, K., Ugrappa, S., & Meka, N. (2016). Photodynamic Therapy—A Non-invasive Treatment Modality for Precancerous Lesions. Journal of lasers in medical sciences, 7(1), 30–36. doi: 10.15171/jlms.2016.07
10	Han et al.	2019	Retrospective study	–	OL	ALA	20%	He–Ne laser	–	632 nm	500 mW/cm^2^	90–180 J/cm^2^	–	≤3	–	3 months	An 86.2% overall response rate was observed in the study, with complete remission at 55.2% and partial remission at 31.0%. ALA-PDT is effective in treating oral leukoplakia, especially when dysplasia is present.	Han, Y., Xu, S., Jin, J., Wang, X., Liu, X., Hua, H., Wang, X., & Liu, H. (2019). Primary Clinical Evaluation of Photodynamic Therapy With Oral Leukoplakia in Chinese Patients. Frontiers in physiology, 9, 1911. doi: 10.3389/fphys.2018.01911
11	Song et al.	2024	Retrospective study	Group 1: “Complete PDT course” group Group 2: “Incomplete PDT course” group Group 3: “No PDT” group	OL	ALA	20%	LED	After each irradiation of 3 min, a 3 min interval was given	635 ± 5 nm	100 mW/cm^2^	100 J/cm^2^	–	–	2–3 weeks	2–185 (mean, 51.7) months	The risk of malignant transformation of OL can be significantly reduced by a complete PDT course, especially in patients who have risk factors for malignant transformation.	Song, Y., Tang, F., Liu, J., Yang, D., Wang, J., Luo, X., Zhou, Y., Zeng, X., Xu, H., Chen, Q., & Dan, H. (2024). A complete course of photodynamic therapy reduced the risk of malignant transformation of oral leukoplakia. Photodiagnosis and photodynamic therapy, 49, 104338. doi: 10.1016/j.pdpdt.2024.104338
12	Wang et al.	2024	Retrospective study	–	OL	ALA	20%	He–Ne laser	–	635 nm	150–300 mW/cm^2^	–	300 s	3–4	7–14 days	2 years	ALA-PDT is considered an effective option for treating OLK, notably for homogenous leukoplakia, with few adverse effects.	Wang, Y., Tang, H., Wang, K., Zhao, Y., Xu, J., & Fan, Y. (2024). Clinical evaluation of photodynamic therapy for oral leukoplakia: a retrospective study of 50 patients. BMC oral health, 24(1), 9. doi: 10.1186/s12903-023-03791-5
13	Yao et al.	2022	Retrospective study	–	OL	ALA (pretreatment: AFL)	20%	LED	–	630 nm	–	180 J/cm^2^	5 min	1	–	3 years	AFL-PDT is an effective management of OL, but it requires consistent follow-up.	Yao, Y. L., Wang, Y. F., Li, C. X., Wu, L., & Tang, G. Y. (2022). Management of oral leukoplakia by ablative fractional laser-assisted photodynamic therapy: A 3-year retrospective study of 48 patients. Lasers in surgery and medicine, 54(5), 682–687. doi: 10.1002/lsm.23534
14	Ou et al.	2022	Clinical trial	–	OL	ALA (pretreatment: YSGG)	20%	LED	–	635 nm	80 mW/cm^2^	120 J/cm^2^	25 min	–	–	1 years	60 cases (84.51%) experienced complete remission, and 11 cases (15.49%) experienced partial remission. Using ALA-mediated PDT together with YSGG provides a new strategy for OL treatment.	Ou, J., Gao, Y., Li, H., Ling, T., & Xie, X. (2022). Application of 5-aminolevulinic acid-mediated Waterlase-assisted photodynamic therapy in the treatment of oral leukoplakia. Scientific reports, 12(1), 9391. doi: 10.1038/s41598-022-13497-3
15	Pietruska et al.	2014	Clinical trial	–	OL	chlorin-e6 (Photolon®)	20%	Semiconductor laser	–	660 nm	<300 mW/cm^2^	90 J/cm^2^	–	10	2 weeks	No follow up	Chlorine-e6 mediated PDT can significantly reduce the size of OL lesions and might be beneficial in clinical practice.	Pietruska, M., Sobaniec, S., Bernaczyk, P., Cholewa, M., Pietruski, J. K., Dolińska, E., Skurska, A., Duraj, E., & Tokajuk, G. (2014). Clinical evaluation of photodynamic therapy efficacy in the treatment of oral leukoplakia. Photodiagnosis and photodynamic therapy, 11(1), 34–40. doi: 10.1016/j.pdpdt.2013.10.003
16	Rabinovich et al.	2022	Retrospective study	–	OL	Photoditazine	0.5%	LED	–	661–668 nm	0.4 W	100–150 J/cm^2^	10–30 min	–	–	3 years	PDT is an effective treatment method for patients with complex forms of leukoplakia.	Rabinovich, O. F., Rabinovich, I. M., Umarova, K. V., Shindich, O. I., & Kozhedub, A. A. (2022). Primenenie fotodinamicheskoi terapii u patsientov s tyazhelymi formami leikoplakii The use of photodynamic therapy in patients with severe forms of leukoplakia. Stomatologiia, 101(5), 11–16. doi: 10.17116/stomat202210105111
17	Yu et al.	2009	Prospective study	Group 1: ALA-PDT using LED light Group 2: ALA-PDT using Laser light	OEL	ALA	20%	LED	Five 3-min and one 100 s irradiations	630 ± 5 nm	100 mW/cm^2^	100 J/cm^2^	1,000 s	≤8 (mean, 3.5)	1 week	16–76 (mean, 32) months	No differences were observed between groups. ALA-PDT using either the LED or laser light is an effective treatment modality for OEL lesions.	Yu, C. H., Lin, H. P., Chen, H. M., Yang, H., Wang, Y. P., & Chiang, C. P. (2009). Comparison of clinical outcomes of oral erythroleukoplakia treated with photodynamic therapy using either light-emitting diode or laser light. Lasers in surgery and medicine, 41(9), 628–633. doi: 10.1002/lsm.20841
								Laser light		635 nm	100 mW/cm^2^	100 J/cm^2^	1,000 s			3–16 (mean, 10) months		
18	Lin et al.	2010	Prospective study	–	OEL	ALA	20%	Arts-Laser diode laser	Five 3-min and one 100 s irradiations	635 nm	100 mW/cm^2^	100 J/cm^2^	1,000 s	≤8	1 week	6–30 (mean, 18) months	ALA-PDT is very effective for OVH and OEL lesions.	Lin, H. P., Chen, H. M., Yu, C. H., Yang, H., Wang, Y. P., & Chiang, C. P. (2010). Topical photodynamic therapy is very effective for oral verrucous hyperplasia and oral erythroleukoplakia. Journal of oral pathology & medicine: official publication of the International Association of Oral Pathologists and the American Academy of Oral Pathology, 39(8), 624–630. doi: 10.1111/j.1600-0714.2010.00935.x
					OVH											8–37 (mean, 20) months		
19	Chen et al.	2004	Clinical trial	–	OVH	ALA	5%	LED	Five 3 min and one 100 s irradiations separated by five 3 min rests	635 ± 5 nm	100 mW/cm^2^	100 J/cm^2^	1,000 s	1–3	1 week	3–11 (mean, 5.6) months	Complete lesion regression was achieved with 1–3 ALA-PDT treatments.	Chen, H. M., Chen, C. T., Yang, H., Kuo, M. Y., Kuo, Y. S., Lan, W. H., Wang, Y. P., Tsai, T., & Chiang, C. P. (2004). Successful treatment of oral verrucous hyperplasia with topical 5-aminolevulinic acid-mediated photodynamic therapy. Oral oncology, 40(6), 630–637. doi: 10.1016/j.oraloncology.2003.12.010
20	Yu et al.	2008	Clinical trial	–	OVH	ALA	20%	LED	Five 3-min and one 100 s irradiations	630 ± 5 nm	100 mW/cm^2^	100 J/cm^2^	1,000 s	3.7 ± 1.5 (Buccal mucosa); 4.1 ± 1.4 (Other oral mucosal)	1 week	6–56 (mean, 26) months	Lesions of OVH that are 3.1 cm or smaller can fully regress with under less than seven weekly applications of ALA-PDT.	Yu, C. H., Chen, H. M., Hung, H. Y., Cheng, S. J., Tsai, T., & Chiang, C. P. (2008). Photodynamic therapy outcome for oral verrucous hyperplasia depends on the clinical appearance, size, color, epithelial dysplasia, and surface keratin thickness of the lesion. Oral oncology, 44(6), 595–600. doi: 10.1016/j.oraloncology.2007.08.016
21	Sulewska et al.	2017	Case series	–	Erosive OLP	ALA	5%	Diode lamp	–	630 nm	300 mW	150 J/cm^2^	500 s	≤ 10	1 week	12 months follow up with 6 appointments: a week, month, and subsequently 3, 6, 9 and 12 months after the final PDT	Photodynamic therapy offers a non-invasive option for treating oral mucosa lesions and might become an alternative or supplement to current treatments.	Sulewska, M., Duraj, E., Sobaniec, S., Graczyk, A., Milewski, R., Wróblewska, M., Pietruski, J., & Pietruska, M. (2017). A clinical evaluation of the efficacy of photodynamic therapy in the treatment of erosive oral lichen planus: A case series. Photodiagnosis and photodynamic therapy, 18, 12–19. doi: 10.1016/j.pdpdt.2017.01.178
22	Rakesh et al.	2018	Case series	One side of mouth treated with ALA-PDT; other side was control	Erosive OLP	ALA	4%	Diode laser	–	600–670 nm	–	80 J/cm^2^	–	1	–	Upto 4 years	PDT might serve as an additional treatment option for OLP lesions that are resistant to symptoms.	Rakesh, N., Clint, J. B., Reddy, S. S., Nagi, R., Chauhan, P., Sharma, S., Sharma, P., Kaur, A., Shetty, B., Ashwini, S., Pavan Kumar, T., & Vidya, G. S. (2018). Clinical evaluation of photodynamic therapy for the treatment of refractory oral Lichen planus—A case series. Photodiagnosis and photodynamic therapy, 24, 280–285. doi: 10.1016/j.pdpdt.2018.09.011
23	Sulewska et al.	2019	Case series	–	Reticular OLP	ALA	5%	Custom-made diode lamp	–	630 nm	300 mW	150 J/cm^2^	–	5–10	1 week	12 months follow up with 6 appointments: a week, month, and subsequently 3, 6, 9 and 12 months after the final PDT	ALA-PDT using a 630 nm light proved effective and can be considered an alternative treatment for symptomatic OLP.	Sulewska, M., Duraj, E., Sobaniec, S., Graczyk, A., Milewski, R., Wróblewska, M., Pietruski, J., & Pietruska, M. (2019). A clinical evaluation of efficacy of photodynamic therapy in treatment of reticular oral lichen planus: A case series. Photodiagnosis and photodynamic therapy, 25, 50–57. doi: 10.1016/j.pdpdt.2018.11.009
24	Sulewska et al.	2023	Case series	–	Reticular OLP	ALA	5%	Diode lamp	–	630 nm	300 mW	120 J/cm^2^ (peak power density)	–	10	1 week	12 months	ALA-PDT showed effectiveness in treating the reticular OLP and might be considered as an optional or complementary treatment.	Sulewska, M. E., Tomaszuk, J., Sajewicz, E., Pietruski, J., Starzyńska, A., & Pietruska, M. (2023). Treatment of Reticular Oral Lichen Planus with Photodynamic Therapy: A Case Series. Journal of clinical medicine, 12(3), 875. doi: 10.3390/jcm12030875
25	Ming et al.	2024	Case series	–	Refractory erosive OLP	ALA	20%	Laser	Repeated 5 times for 3 min and rested for 3 min after each time	630 nm	100 mW/cm^2^	–	15 min	1–2	–	–	PDT could serve as an effective alternative for treating refractory erosive OLP.	Ming, J., Yang, Z., Wang, T., Wang, J., & Zeng, X. (2024). Photodynamic therapy for refractory erosive oral lichen planus: a case series study. Oral surgery, oral medicine, oral pathology and oral radiology, 137(3), e41–e44. doi: 10.1016/j.oooo.2023.11.013
26	Kvaal et al.	2013	Open, nonrandomized, noncomparative prospective study	One side of mouth treated with MAL-PDT; other side was control	OLP	MAL	–	LED	–	600–660 nm	100–130 mW/cm^2^	75 J/cm^2^	–	1	–	6–48 (30, mean) months	OLP treated with MAL-PDT showed sustained improvement after a single treatment.	Kvaal, S. I., Angell-Petersen, E., & Warloe, T. (2013). Photodynamic treatment of oral lichen planus. Oral surgery, oral medicine, oral pathology and oral radiology, 115(1), 62–70. doi: 10.1016/j.oooo.2012.08.448
27	Aghahosseini et al.	2006	Open before-after study	–	Keratotic, atrophic and erosive OLP	Methylene blue	5%	Diode laser	–	632 nm	–	120 J/cm^2^	2 min	–	–	Weekly upto 12 weeks	MB-PDT appears to be a promising alternative therapy for managing OLP.	Aghahosseini, F., Arbabi-Kalati, F., Fashtami, L. A., Djavid, G. E., Fateh, M., & Beitollahi, J. M. (2006). Methylene blue-mediated photodynamic therapy: a possible alternative treatment for oral lichen planus. Lasers in surgery and medicine, 38(1), 33–38. doi: 10.1002/lsm.20278
28	Sadaksharam et al.	2012	Before and after study	–	Reticular and erosive OLP	Methylene blue	5%	Xenon arc lamp	–	632 ± 5 nm	–	120 J/cm^2^	20 min	4	3 or 8 days	2nd and 4th week, and 6 months	MB-PDT might be a promising alternative for the management of oral lichen planus.	Sadaksharam, J., Nayaki, K. P., & Selvam, N. P. (2012). Treatment of oral lichen planus with methylene blue mediated photodynamic therapy–a clinical study. Photodermatology, photoimmunology & photomedicine, 28(2), 97–101. doi: 10.1111/j.1600-0781.2012.00647.x
29	Mostafa et al.	2017	RCT	Control group: conventional topical corticosteroids (TC) treatment Study group: PDT	Erosive OLP	Methylene blue	5%	Blue diode laser	–	660 nm	100–130 mW/cm^2^	–	–	8	1 week	2 months	MB-PDT is regarded as a superior treatment for erosive OLP compared to TC due to its greater effectiveness in reducing pain and regressing lesions.	Mostafa, D., Moussa, E., & Alnouaem, M. (2017). Evaluation of photodynamic therapy in treatment of oral erosive lichen planus in comparison with topically applied corticosteroids. Photodiagnosis and photodynamic therapy, 19, 56–66. doi: 10.1016/j.pdpdt.2017.04.014
30	Bakhtiari et al.	2017	RCT	Control group: topical corticosteroid (0.1 mg/ml dexamethasone) Study group: PDT	Reticular and erosive OLP	Methylene blue	5%	LED	–	630 nm	–	7.2–14.4 J/cm^2^	30–120 s	4	3 or 7 days	3 months	PDT was as effective as dexamethasone mouthwash, and could be used as a safe treatment option for OLP, with no definite side effects.	Bakhtiari, S., Azari-Marhabi, S., Mojahedi, S. M., Namdari, M., Rankohi, Z. E., & Jafari, S. (2017). Comparing clinical effects of photodynamic therapy as a novel method with topical corticosteroid for treatment of Oral Lichen Planus. Photodiagnosis and photodynamic therapy, 20, 159–164. doi: 10.1016/j.pdpdt.2017.06.002
31	Saleh et al.	2022	Before and after study	–	Erosive OLP of type 2 diabetic and hypertensive patients	Methylene blue	–	–	–	660 nm	100–130 mW/cm^2^	–	–	8	0.5 week	2 weeks and one month	PDT was successful and well-received by patients, helping to prevent the effects of systemic corticosteroids in individuals with diabetes and high blood pressure.	Saleh, W., & Khashaba, O. (2022). Clinical responses of patients with systemic diseases to the photodynamic therapy of oral lichen planus. Photodiagnosis and photodynamic therapy, 39, 102972. doi: 10.1016/j.pdpdt.2022.102972
32	Salinas-Gilabert et al.	2022	RCT	Group 1: PDT + orabase cream Group 2: low-power laser + orabase cream Group 3: inactive laser + 0.1% triamcinolone acetonide	OLP	Methylene blue	1%	Helbo® Theralite Laser	–	–	200 mW/cm^2^	6 J/cm^2^	30 s	4	1 week	3 months	PDT and photobiomodulation, applied weekly for four weeks, offer safe and non-invasive treatment options, with the important advantage of lacking adverse effects.	Salinas-Gilabert, C., Gómez García, F., Galera Molero, F., Pons-Fuster, E., Vander Beken, S., & Lopez Jornet, P. (2022). Photodynamic Therapy, Photobiomodulation and Acetonide Triamcinolone 0.1% in the Treatment of Oral Lichen Planus: A Randomized Clinical Trial. Pharmaceutics, 15(1), 30. doi: 10.3390/pharmaceutics15010030
33	Jajarm et al.	2015	RCT	Control group: topical corticosteroid (0.1 mg/ml dexamethasone) Study group: PDT	Erosive, atrophic OLP	Toluidine blue	1 mg/ml	GaAlAs laser	–	630 nm	10 mW/cm^2^	1.5 J/cm^2^	2.5 min	2	0.5 week	1 month	TB-PDT offers an effective treatment and can be considered an alternative for erosive-atrophic OLP. However, it should be highlighted that traditional corticosteroid treatment produced better results than TB-PDT.	Jajarm, H. H., Falaki, F., Sanatkhani, M., Ahmadzadeh, M., Ahrari, F., & Shafaee, H. (2015). A comparative study of toluidine blue-mediated photodynamic therapy versus topical corticosteroids in the treatment of erosive-atrophic oral lichen planus: a randomized clinical controlled trial. Lasers in medical science, 30(5), 1475–1480. doi: 10.1007/s10103-014-1694-1
34	Mirza et al.	2018	RCT	Group 1: PDT Group 2: Low Level Laser Therap (LLLT) Group 3: Topical 0.1 mg/ml dexamethasone	Erosive-atrophic OLP	Toluidine blue	1 mg/ml	GaAlAs laser	–	630 nm	10 mW/cm^2^	1.5 J/cm^2^	2.5 min	2	0.5 week	1 year	PDT and LLLT are successful in treating erosive-atrophic OLP in adults.	Mirza, S., Rehman, N., Alrahlah, A., Alamri, W. R., & Vohra, F. (2018). Efficacy of photodynamic therapy or low level laser therapy against steroid therapy in the treatment of erosive-atrophic oral lichen planus. Photodiagnosis and photodynamic therapy, 21, 404–408. doi: 10.1016/j.pdpdt.2018.02.001
35	Lavaee and Shadmanpour et al.	2019	RCT	Control group: topical corticosteroid (1% triamcinolone acetonide) Study group: PDT	Bilateral oral OLP	Toluidine blue	1 mg/ml	Diode laser InGaAlP	–	660 nm	25 mW/cm^2^	19.23 J/cm^2^	10 min	3	1 week	7 weeks	PDT can serve as an alternative treatment in conjunction with standard approaches or as a novel option for resistant OLP.	Lavaee, F., & Shadmanpour, M. (2019). Comparison of the effect of photodynamic therapy and topical corticosteroid on oral lichen planus lesions. Oral diseases, 25(8), 1954–1963. doi: 10.1111/odi.13188
36	Romano et al.	2019	Before and after study	–	OLP	Toluidine blue	–	FotoSan® 630	–	630 nm	–	–	–	≤5	≥14 days	No follow up	TB-PDT caused the complete disappearance of clinically visible lesions in 4 out of 5 cases.	Romano, A., Contaldo, M., Della Vella, F., Russo, D., Lajolo, C., Serpico, R., & Di Stasio, D. (2019). Topical toluidine blue-mediated photodynamic therapy for the treatment of oral lichen planus. Journal of biological regulators and homeostatic agents, 33(3 Suppl. 1).
37	Sikdar et al.	2022	Prospective interventional study	Group 1: 0.1% Triamcinolone acetonide Group 2: PDT	Erosive OLP	Toluidine blue	1 mg/ml	Diode laser	Continuous wave in three cycles, 3 min/cycle	980 nm	0.1 W	54 J/cm^2^	9 min	8	0.5 week	4 weeks	PDT provides better treatment for erosive lichen planus and can serve as an alternative to conventional treatment methods.	Sikdar, A., S. PramodKumar, J. Pathi, S. N. Chinnannavar, D. K. Singh and S. Jha (2022). “Computerised Photometric Analysis of Photodynamic Therapy versus Triamcinolone Acetonide for Treatment of Erosive Lichen Planus—A Prospective Interventional Study.” Journal of Clinical and Diagnostic Research 16(4): ZC40–ZC44.
38	Sobaniec et al.	2013	Before and after clinical study	–	OLP	Chlorin-e6-Photolon®	20%	Semiconductor laser	–	660 nm	≤300 mW	90 J/cm^2^	–	≤10	2 weeks	No follow up	A mean reduction of 55% in lesions from the study suggests PDT could be an alternative therapy for OLP.	Sobaniec, S., Bernaczyk, P., Pietruski, J., Cholewa, M., Skurska, A., Dolińska, E., Duraj, E., Tokajuk, G., Paniczko, A., Olszewska, E., & Pietruska, M. (2013). Clinical assessment of the efficacy of photodynamic therapy in the treatment of oral lichen planus. Lasers in medical science, 28(1), 311–316. doi: 10.1007/s10103-012-1153-9
39	Rabinovich et al.	2016	Comparative study	Group 1: Conventional treatment Group 2: Conventional treatment + PDT Group 3: PDT	erosive and ulcerative OLP	Photoditazine	–	Laser Alod-01	–	662 nm	0.568–0.795 W/cm^2^	280 J/cm^2^	–	3–5	–	No follow up	PDT could be an effective method in the intricate management of severe oral lichen planus.	Rabinovich, O. F., Rabinovich, I. M., & Guseva, A. V. (2016). Lechenie patsientov s tyazhelymi formami krasnogo ploskogo lishaya slizistoi obolochki rta s primeneniem fotodinamicheskoi terapii Photodynamic therapy in treatment of severe oral lichen planus. Stomatologiia, 95(4), 27–30. doi: 10.17116/stomat201695427-30
40	Fan et al.	1996	Prospective clinical study	–	Moderate to severe dysplasia or SCC	ALA	60 mg/kg	Gold vapor laser	–	628 nm	＜250 mW/cm^2^	100 or 200 J/cm^2^	–	–	–	6–18 months	ALA-PDT provide a simple and effective treatment approach for dysplasia of the mouth. Outcomes in invasive cancers are not as favorable, primarily because the PDT effect is too superficial with the current treatment protocols using ALA as the photosensitizer.	Fan, K. F., Hopper, C., Speight, P. M., Buonaccorsi, G., MacRobert, A. J., & Bown, S. G. (1996). Photodynamic therapy using 5-aminolevulinic acid for premalignant and malignant lesions of the oral cavity. Cancer, 78(7), 1374–1383. doi: 10.1002/(SICI)1097-0142(19961001)78:7<1374::AID-CNCR2>3.0.CO;2-L
41	Jerjes et al.	2011	Prospective clinical study	–	Thin mild-moderate dysplasia	ALA	60 mg/kg	Diode laser	–	628 nm	–	100 or 200 J/cm^2^	–	–	–	mean 7.3 years	ALA-PDT and/or mTHPC-PDT provide an effective alternative therapy for oral potentially malignant disorders.	Jerjes, W., Upile, T., Hamdoon, Z., Mosse, C. A., Akram, S., & Hopper, C. (2011). Photodynamic therapy outcome for oral dysplasia. Lasers in surgery and medicine, 43(3), 192–199. doi: 10.1002/lsm.21036
					Thicker mild-moderate dysplasia, severe dysplasia and carcinoma in situ	mTHPC	0.1 mg/kg	Diode laser	–	652 nm	–	20 J/cm^2^	–					
42	Ahn et al.	2016	Phase 1 trial	Group 1: Low (50 and 100 J/cm^2^) fluence groups Group 2: High (150 and 200 J/cm^2^) fluence groups	High-grade dysplasia, carcinoma in situ, or microinvasive (≤1.5 mm depth) SCC	ALA	60 mg/kg	Diode laser	continuous (unfractionated) or fractionated (two-part) illumination	629–635 nm	100 mW/cm^2^	50, 100, 150 or 200 J/cm^2^	–	–	–	3.2–59.4 (41.6, mean) months	ALA-PDT is safe with high-grade dysplasia, carcinoma in situ and early head and early stage carcinomas of the head and neck, and the side effects are usually tolerable.	Ahn, P. H., Quon, H., O'Malley, B. W., Weinstein, G., Chalian, A., Malloy, K., Atkins, J. H., Sollecito, T., Greenberg, M., McNulty, S., Lin, A., Zhu, T. C., Finlay, J. C., Cengel, K., Livolsi, V., Feldman, M., Mick, R., & Busch, T. M. (2016). Toxicities and early outcomes in a phase 1 trial of photodynamic therapy for premalignant and early stage head and neck tumors. Oral oncology, 55, 37–42. doi: 10.1016/j.oraloncology.2016.01.013
43	Sotiriou et al.	2011	Prospective clinical trial	-	AC	MAL	16%	Waldmann PDT 1200	–	570–670 nm	80 mW/cm^2^	40 J/cm^2^	–	2	2 weeks	12 months	Sequential use of PDT and imiquimod cream is highly beneficial for treating AC.	Sotiriou, E., Lallas, A., Goussi, C., Apalla, Z., Trigoni, A., Chovarda, E., & Ioannides, D. (2011). Sequential use of photodynamic therapy and imiquimod 5% cream for the treatment of actinic cheilitis: a 12-month follow-up study. The British journal of dermatology, 165(4), 888–892. doi: 10.1111/j.1365-2133.2011.10478.x
44	Fai et al.	2012	Retrospective case series	–	AC	MAL	16%	LED	–	–	–	37 J/cm^2^	–	1–2	1 week	6–36 (20, mean) months	MAL-PDT could be viewed as a novel and effective treatment for AC, providing excellent cosmetic results and long-lasting clinical benefits for most patients.	Fai, D., Romano, I., Cassano, N., & Vena, G. A. (2012). Methyl-aminolevulinate photodynamic therapy for the treatment of actinic cheilitis: a retrospective evaluation of 29 patients. Giornale italiano di dermatologia e venereologia: organo ufficiale, Societa italiana di dermatologia e sifilografia, 147(1), 99–101.
45	Ribeiro et al.	2012	Experimental, non controlled clinical trial	–	AC	MAL	16%	LED	–	630 nm	71 mW/cm^2^	37 J/cm^2^	8 min and 40 s	1	–	51–94 (62.5, mean) days	PDT serves as an effective treatment for actinic cheilitis, though it is often accompanied by significant pain.	Ribeiro, C. F., Souza, F. H., Jordão, J. M., Haendchen, L. C., Mesquita, L., Schmitt, J. V., & Faucz, L. L. (2012). Photodynamic therapy in actinic cheilitis: clinical and anatomopathological evaluation of 19 patients. Anais brasileiros de dermatologia, 87(3), 418–423. doi: 10.1590/s0365-05962012000300011
46	Kim et al.	2013	Prospective study	–	AC	MAL	–	Aktilite CL 128	–	–	–	37 J/cm^2^	–	4.30 ± 1.89	2–4 weekls	2–45 months	The first cure rate of PDT was 50.0% and the final cure rate was 30.0%.	Kim, S. K., Song, H. S., & Kim, Y. C. (2013). Topical photodynamic therapy may not be effective for actinic cheilitis despite repeated treatments. European journal of dermatology: EJD, 23(6), 917–918. doi: 10.1684/ejd.2013.2199
47	Choi et al.	2015	RCT	Group 1: Er:YAG AFL MAL-PDT Group 2: MAL-PDT	AC	MAL	16%	LED	–	632 nm	–	37 J/cm^2^	–	Group 1: 1; Group 2: 2	–	1 week, 3 and 12 months	One session of Er:YAG AFL MAL-PDT was more effective and resulted in a lower recurrence rate compared to two sessions of MAL-PDT for treating AC lesions.	Choi, S. H., Kim, K. H., & Song, K. H. (2015). Efficacy of ablative fractional laser-assisted photodynamic therapy for the treatment of actinic cheilitis: 12-month follow-up results of a prospective, randomized, comparative trial. The British journal of dermatology, 173(1), 184–191. doi: 10.1111/bjd.13542
48	Fai et al.	2015	Retrospective case series	–	AC	MAL	16%	Daylight	–	–	–	–	2 h	2	1–2 weeks	6–12 months	D-PDT presents an appealing option for the treatment of AC. D-PDT has relevant advantages over conventional PDT, such as a simpler procedure and generally good tolerability.	Fai, D., Romanello, E., Brumana, M. B., Fai, C., Vena, G. A., Cassano, N., & Piaserico, S. (2015). Daylight photodynamic therapy with methyl-aminolevulinate for the treatment of actinic cheilitis. Dermatologic therapy, 28(6), 355–368. doi: 10.1111/dth.12258
49	Suárez-Pérez et al.	2015	Prospective study	–	AC	MAL	16%	LED	1st dose 20 J/cm^2^, 2 h later 80 J/cm^2^	630 nm	–	100 J/cm^2^	–	1	–	3–18 months	Considering the response rates from both clinical and histological perspectives, PDT cannot be deemed a first-line treatment for AC. However, given its excellent cosmetic outcomes and non-invasive characteristics, it could be a compelling option in selected cases.	Suárez-Pérez, J. A., López-Navarro, N., Herrera-Acosta, E., Aguilera, J., Gallego, E., Bosch, R., & Herrera, E. (2015). Treatment of actinic cheilitis with methyl aminolevulinate photodynamic therapy and light fractionation: a prospective study of 10 patients. European journal of dermatology: EJD, 25(6), 623–624. doi: 10.1684/ejd.2015.2648
50	Chaves et al.	2017	Prospective study	–	AC	MAL	–	Aktilite®	–	–	71 mW/cm^2^	37 J/cm^2^	8 min	2	2 weeks	3 months	In this trial, photodynamic therapy was not an efficacious therapeutic treatment for patients with actinic cheilitis in this sample.	Chaves, Y. N., Torezan, L. A., Lourenço, S. V., & Neto, C. F. (2017). Evaluation of the efficacy of photodynamic therapy for the treatment of actinic cheilitis. Photodermatology, photoimmunology & photomedicine, 33(1), 14–21. doi: 10.1111/phpp.12281
51	Levi et al.	2019	Retrospective study	–	AC	MAL	–	Daylight	–	–	–	–	2.5 h	1–6	2–4 weeks	6–60 (30, mean) months	Daylight photodynamic therapy is emerging as a promising treatment for actinic cheilitis.	Levi, A., Hodak, E., Enk, C. D., Snast, I., Slodownik, D., & Lapidoth, M. (2019). Daylight photodynamic therapy for the treatment of actinic cheilitis. Photodermatology, photoimmunology & photomedicine, 35(1), 11–16. doi: 10.1111/phpp.12415
52	Andreadis et al.	2020	Prospective study	–	AC	MAL	16%	Daylight	–	–	–	–	2 h	2	1 week	3, 6, and 12 months	DLPDT appears to offer considerable benefit in treating grade I AC. Combining with other treatment approaches could boost the effectiveness in grade II AC.	Andreadis, D., Pavlou, A., Vakirlis, E., Anagnostou, E., Vrani, F., Poulopoulos, A., Kolokotronis, A., Ioannidis, D., & Sotiriou, E. (2020). Daylight photodynamic therapy for the management of actinic cheilitis. Archives of dermatological research, 312(10), 731–737. doi: 10.1007/s00403-020-02069-y
53	Arisi et al.	2022	RCT	Group 1: Conventional PDT	AC	MAL	16%	Aktilite CL128	–	630 ± 5 nm	–	–	–	1–2	–	3 months	Indoor daylight PDT presents a viable option to Conventional PDT for AC treatment, with superior tolerability and lack of inferior efficacy.	Arisi, M., Galli, B., Pisani, E. G., La Rosa, G., Licata, G., Rovaris, S., Tomasi, C., Rossi, M., Venturini, M., Spiazzi, L., & Calzavara-Pinton, P. (2022). Randomized Clinical Trial of Conventional versus Indoor Daylight Photodynamic Therapy for Treatment of Actinic Cheilitis. Dermatology and therapy, 12(9), 2049–2061. doi: 10.1007/s13555-022-00783-1
				Group 2: Indoor daylight PDT		MAL	16%	Polychromatic white LED lamp	–	400–700 nm	–	–	2 h	1–2	–			
54	Berking et al.	2007	Prospective, uncontrolled study	–	AC	ALA (MAOP)	–	–	–	630 nm	–	37 J/cm^2^	–	2	1 week	Upto 22 months	PDT offers a different treatment choice for patients with actinic cheilitis, particularly for those at greater risk from invasive therapies.	Berking, C., Herzinger, T., Flaig, M. J., Brenner, M., Borelli, C., & Degitz, K. (2007). The efficacy of photodynamic therapy in actinic cheilitis of the lower lip: a prospective study of 15 patients. Dermatologic surgery: official publication for American Society for Dermatologic Surgery [et al.], 33(7), 825–830. doi: 10.1111/j.1524-4725.2007.33176.x
55	Sotiriou et al.	2010	Prospective clinical trial	–	AC	ALA	20%	Waldmann PDT 1200	–	570–670 nm	80 mW/cm^2^	40 J/cm^2^	–	2	2 weeks	18 months	PDT represents a moderately effective treatment modality in AC. Further refinement of treatment procedures and protocols is necessary to achieve higher response rates.	Sotiriou, E., Apalla, Z., Chovarda, E., Panagiotidou, D., & Ioannides, D. (2010). Photodynamic therapy with 5-aminolevulinic acid in actinic cheilitis: an 18-month clinical and histological follow-up. Journal of the European Academy of Dermatology and Venereology: JEADV, 24(8), 916–920. doi: 10.1111/j.1468-3083.2009.03550.x
56	Radakovic and Tanew	2017	Retrospective study	–	AC	ALA	–	LED	–	630 ± 9 nm	61.7 mW/cm^2^	37 J/cm^2^	10 min	1–2	1–2 weeks	12 months	PDT is an efficient and easy to perform therapeutic modality for AC on both the lower and upper lips.	Radakovic, S., & Tanew, A. (2017). 5-aminolaevulinic acid patch-photodynamic therapy in the treatment of actinic cheilitis. Photodermatology, photoimmunology & photomedicine, 33(6), 306–310. doi: 10.1111/phpp.12332

AC,actinic cheilitis; ALA, 5-aminolevulinic acid; AFL, ablative fractional laser; cm^2^, square centimeter; MAL, methyl aminolevulinate; MAOP, methylaminoxopentanoate (methylated ester of 5-ALA); min, minutes; mTHPC, meso-tetrahydroxyphenyl chlorin (meta-tetrahydroxy-phenyl chlorin); nm, nanometers; mW, milliwatts; OL, oral leukoplakia; OLP, oral lichen planus; OEL, oral erythroleukoplakia; OVH, oral verrucous hyperplasia; RCT, randomized clinical trial; s, seconds; SCC, squamous cell carcinoma; YSGG, Waterlase; -, not reported.

## 4 Periodontitis

Periodontitis is a chronic multi-factorial inflammatory disease brought on by bacteria and is characterized by the loss of alveolar bone and loosening of the teeth (29, 30); it is related to the formation of the dental plaque biofilm. Mechanical debridement (MD) and antibiotic treatment are frequently utilized in clinics. However, mechanical debridement frequently leaves dental plaque in periodontal pockets, furcations, and uneven root surface areas, ultimately leading to treatment failure. *Porphyromonas gingivalis* (*P. gingivalis*) and *Aggregatibacter actinomycetemcomitans* (*A. actinomycetemcomitans*) are primarily responsible for periodontitis (31). The effectiveness of many systemic antimicrobials is constrained because they cannot continuously suppress *P. gingivalis* and *A. actinomycetemcomitans*.

Antimicrobial resistance, dysbacteriosis, and gastrointestinal disorders are increasingly common due to antibiotic abuse, resulting in antibiotic failures; therefore, a more effective method for treating periodontitis is needed. PDT may be helpful in hard-to-reach places like periodontal pockets or furcation sites for microbial reduction without bacterial resistance, demonstrating its potential as an adjuvant treatment for periodontitis (Table 3). Besides having a bactericidal effect on the periodontal tissues, PDT has an anti-inflammatory effect by lowering inflammatory mediator levels, creating a more favorable healing environment, and re-establishing the cellular biological balance (32).

**Table 3:** Summary of studies on PDT for the treatment of periodontitis.

	Study	Year	Study design	Treatment groups	Lesion type	Type of photosensitizers	Photosensitizers dose	Light source	Irradiation mode	Wave length	Fluence rate/Power	Energy density	Irradiation time	Treatment sessions	Treatment interval	Microorganisms	Follow up	Primary outcome	Referrence
1	Srikanth et al.	2015	RCT	Study group 1: SRP + PDT Study group 2: SRP + laser without PS Control group: SRP	Chronic periodontitis	Indocyanine green	5 mg/ml	Diode laser	Continuous wave mode	810 nm	0.7 W	–	5 s	1	–	*Prevotella intermedia, Veillonella parvula, Fusobacterium nucleatum, Porphyromonas gingivalis,* and *Aggregatibacter actinomycetemcomitans*	1 week, 3 and 6 months	PDT led to a marked reduction in the percentage of viable bacteria at 1 week compared to other groups. No significant differences were observed between CAL and PPD in PDT sites at the end of study period.	Srikanth, K., Chandra, R. V., Reddy, A. A., Reddy, B. H., Reddy, C., and Naveen, A. (2015). Effect of a single session of antimicrobial photodynamic therapy using indocyanine green in the treatment of chronic periodontitis: a randomized controlled pilot trial. Quintessence Int. 46, 391–400. doi: 10.3290/j.qi.a33532
2	Monzavi et al.	2016	RCT	Study group: SRP + PDT Control group: SRP	Chronic periodontitis	Indocyanine green	1 mg/ml	Diode laser	Continuous mode	810 nm	200 mW	–	10 s	4	7 or 10 days	–	1 and 3 months	BOP, PPD and FMBS showed significant improvements in the PDT group. Regarding PI, FMPS and CAL, no significant differences were observed between both groups.	Monzavi, A., Chinipardaz, Z., Mousavi, M., Fekrazad, R., Moslemi, N., Azaripour, A., et al. (2016). Antimicrobial photodynamic therapy using diode laser activated indocyanine green as an adjunct in the treatment of chronic periodontitis: a randomized clinical trial. Photodiagnosis Photodyn. Ther. 14, 93–97. doi: 10.1016/j.pdpdt.2016.02.007
3	Hill et al.	2019	RCT	Study group: SRP + PDT Control group: SRP	Chronic periodontitis	Indocyanine green	0.1 mg/ml	Diode laser	Pulse repetition rate: 2 kHz	808 nm	100 mW	–	20 s	1	–	*Aggregatibacter actinomycetemcomitans, Porphyromonas gingivalis, Prevotella intermedia, Tannerella forsythia, and Treponema denticola*	2 weeks, 3 and 6 months	Median values for BOP, RAL, PD, decreased significantly in both groups at 3 months without significant difference between the groups. PDT significantly decreased the sulcus fluid flow rate (SFFR) at 2 weeks.	Hill, G., Dehn, C., Hinze, A. V., Frentzen, M., and Meister, J. (2019). Indocyanine green-based adjunctive antimicrobial photodynamic therapy for treating chronic periodontitis: a randomized clinical trial. Photodiagnosis Photodyn. Ther. 26, 29–35. doi: 10.1016/j.pdpdt.2019.02.019
4	Joshi et al.	2020	RCT	Study group: SRP + PDT Control group: SRP	Chronic periodontitis	Indocyanine green	1 mg/ml	Diode laser	–	810 nm	200 mW	–	30 s	1	–	–	3 months	There was a significant reduction in PI and mSBI f in both the groups. PDT led to a notable enhancement in PPD and CAL when compared to control group.	Joshi, K., Baiju, C. S., Khashu, H., and Bansal, S. (2020). Clinical effectiveness of indocyanine green mediated antimicrobial photodynamic therapy as an adjunct to scaling root planing in treatment of chronic periodontitis- a randomized controlled clinical trial. Photodiagnosis Photodyn. Ther. 29:101591. doi: 10.1016/j.pdpdt.2019.101591
5	Niazi et al.	2020	RCT	Study group 1: SRP + PDT Study group 2: SRP + SP gel Study group 3: SRP	Chronic periodontitis	Indocyanine green	–	GaAlAs diode laser	–	810 nm	100 mW	–	60 s	1	–	–	3 and 6 months	Both PDT and SP treatments contributed to the reduction of periodontal inflammation. PDT showed a notable improvement in clinical attachment level, while SP notably decreased bleeding levels.	Niazi, F. H., Noushad, M., Tanvir, S. B., Ali, S., Al-Khalifa, K. S., Qamar, Z., et al. (2020). Antimicrobial efficacy of indocyanine green-mediated photodynamic therapy compared with Salvadora persica gel application in the treatment of moderate and deep pockets in periodontitis. Photodiagnosis Photodyn. Ther. 29:101665. doi: 10.1016/j.pdpdt.2020.101665
6	Al-Momani	2021	RCT	Group 1: Root surface debridement (RSD) Group 2: ICG-PDT + RSD	Chronic periodontitis	Indocyanine green	0.5 mg/ml	Diode laser	Continuous mode	810 nm	200 mW	4 J/cm^2^	Papilla for 30 s followed by periodontal pocket depth for 10 s	1	–	*Porphyromonas gingivalis, Tannerella forsythia*	3 and 6 months	ICG-PDT significantly improved clinical and antimicrobial parameters in well-controlled and poorly-controlled T2DM having stage III and grade C periodontitis.	Al-Momani, M. M. (2021). Indocyanine-mediated antimicrobial photodynamic therapy promotes superior clinical effects in stage III and grade C chronic periodontitis among controlled and uncontrolled diabetes mellitus: a randomized controlled clinical trial. Photodiagnosis Photodyn. Ther. 35:102379. doi: 10.1016/j.pdpdt.2021.102379
7	AlSarhan et al.	2021	RCT	Study group: SRP + PDT Control group: SRP	Chronic periodontitis	Indocyanine green	0.1 mg/ml	Diode laser	Pulsed wave mode: pulse repetition rate of 2 kHz	808 nm	300 mW	1414.7 J/cm^2^	–	3	1 week	23 bacterial species	Baseline, 1 and 3 months	There was a marked improvements in both periodontal clinical parameters and microbial burden in the PDT group.	AlSarhan, M. A., Altammami, M. A., Alaqeely, R. S., AlEbdi, A., Jasser, R. A., Otaibi, D. A., et al. (2021). Short-term improvement of clinical parameters and microbial diversity in periodontitis patients following Indocyanine green-based antimicrobial photodynamic therapy: a randomized single-blind split-mouth cohort. Photodiagnosis Photodyn. Ther. 35. doi: 10.1016/j.pdpdt.2021.102349
8	Wadhwa et al.	2021	RCT	Study group: SRP + PDT Control group: SRP	Chronic periodontitis	Indocyanine green	250 μg/ml	GaAlAs diode laser	Continuous wave motion	810 nm	500 mW	–	5 s	1	–	–	3 and 6 months	The experimental sites showed a marked improvement in all clinical and microbiological parameters studied at the conclusion of 3 and 6 months of treatment.	Wadhwa, A., Mallapragada, S., and Sharma, P. (2021). Novel indocyanine green mediated antimicrobial photodynamic therapy in the management of chronic periodontitis—a randomized controlled clinico-microbiological pilot study. J. Oral Biol. Craniofac. Res. 11, 57–62. doi: 10.1016/j.jobcr.2020.11.005
9	Sufaru et al.	2022	RCT	Study group: SRP + PDT Control group: SRP	Chronic periodontitis	Indocyanine green	5 mg/ml	Diode laser	Continuous wave mode	810 nm	0.2 W	12 J/cm^2^	60 s	4	1 week	–	6 months	SRP + PDT group produced more significant reductions for BOP, PD and CAL but not for PI and HbA1c, than SRP alone.	Sufaru, I.-G., Martu, M.-A., Luchian, I., Stoleriu, S., Diaconu-Popa, D., Martu, C., et al. (2022). The effects of 810 nm diode laser and Indocyanine green on periodontal parameters and HbA1c in patients with periodontitis and type II diabetes mellitus: a randomized controlled study. Diagnostics 12:1614. doi: 10.3390/diagnostics12071614
10	Annunziata et al.	2023	RCT	Study group: FMUD + PDT Control group: FMUD	Chronic periodontitis	Indocyanine green	1 mg/ml	Diode laser	Pulsed mode (100 ms ON/100 ms OFF)	810 nm	300 mW	–	30 s	2	3 weeks	*Porphyromonas gingivalis, Prevotella intermedia,* *Prevotella nigrescens, Campylobacter rectus, Aggregatibacter actinomycetemcomitans* and *Parvimonas micra*	3 and 6 months	Repeated PDT combined with FMUD offered no benefits other than specific clinical and microbiological improvements compared to FMUD alone.	Annunziata, M., Donnarumma, G., Guida, A., Nastri, L., Persico, G., Fusco, A., Sanz-Sánchez, I., & Guida, L. (2023). Clinical and microbiological efficacy of indocyanine green-based antimicrobial photodynamic therapy as an adjunct to non-surgical treatment of periodontitis: a randomized controlled clinical trial. Clinical oral investigations, 27(5), 2385–2394. doi: 10.1007/s00784-023-04875-w
11	Costa et al.	2023	RCT	Study group: SRP + PDT Control group: SRP	Chronic periodontitis	Indocyanine green	250 μg/ml	Diode laser	Continuous wave mode	909 nm	0.5 W	–	5 s	2	15 days	*P. gingivalis, Aggregatibacter actinomycetemcomitans, Tannerella forsythia, Treponema denticola, Fusobacterium nucleatum,* and *Prevotella intermedia*	Baseline, 3 and 6 months	Significant clinical periodontal improvements were observed in both groups, but with no significant differences between groups except from inflammation parameters.	Costa, F. O., Esteves Lima, R. P., Costa, A. M., Costa, A. A., Mattos Pereira, G. H., Cortelli, S. C., Cortelli, J. R., Magalhães Cyrino, R., Aparecida Silva, T., & Miranda Cota, L. O. (2023). Adjunctive effects of photodynamic therapy using indocyanine green in residual pockets during periodontal maintenance therapy: A split-mouth randomized controlled trial. Journal of periodontology, 94(9), 1100–1111. doi: 10.1002/JPER.22-0672
12	Cetiner et al.	2024	RCT	Study group 1: Adjunctive PDT Study group 2: Adjunctive photobiomodulation Study group 3: Adjunctive ozone Control group: Surgical treatment alone	Chronic periodontitis	Indocyanine green	1 mg/ml	Diode laser	Continuous mode	970 ± 15 nm	2 W	8.6 J/cm^2^	30 s	4	1, 2, 4 days	–	6 months	The application of PDT and LED after regenerative therapy for stage III/IV grade C periodontitis resulted in a notably greater improvement on clinical outcomes in deep periodontal pockets.	Cetiner, D. O., Isler, S. C., Ilikci-Sagkan, R., Sengul, J., Kaymaz, O., & Corekci, A. U. (2024). The adjunctive use of antimicrobial photodynamic therapy, light-emitting-diode photobiomodulation and ozone therapy in regenerative treatment of stage III/IV grade C periodontitis: a randomized controlled clinical trial. Clinical oral investigations, 28(8), 426. doi: 10.1007/s00784-024-05794-0
13	Hayashi et al.	2024	RCT	Study group: PDT Control group: Pseudo PDT	Chronic periodontitis	Indocyanine green	10 mg/ml	Diode laser	100 msec repeated pulse, 50% duty cycle	810 ± 20 nm	1.46 W/cm^2^	250.38 J/cm^2^	3 min	1	–	28 bacterial species	Baseline and 1 week	Immediately following treatment, the study group showed a notable reduction in colony count compared to the count before treatment. Compared to the control group, the study group showed a significantly greater number of patients with colony reduction to ≤50% and ≤10%.	Hayashi, J. I., Ono, K., Iwamura, Y., Sasaki, Y., Ohno, T., Goto, R., Nishida, E., Yamamoto, G., Kikuchi, T., Higuchi, N., Mitani, A., & Fukuda, M. (2024). Suppression of subgingival bacteria by antimicrobial photodynamic therapy using transgingival irradiation: A randomized clinical trial. Journal of periodontology, 95(8), 718–728. doi: 10.1002/JPER.23-0328
14	Betsy et al.	2014	RCT	Study group: SRP + PDT Control group: SRP	Chronic periodontitis	Methylene blue	10 mg/ml	Diode laser	–	655 nm	60 mW/cm^2^	–	60 s	1	–	–	2 weeks, 1, 3 and 6 months	PDT showed a reduction in PPD and CAL at 3 and 6 months, a decrease in PI at 2 weeks, and an improvement in GI and GB at 2 weeks, 1 month, and 3 months compared to group.	Betsy, J., Prasanth, C. S., Baiju, K. V., Prasanthila, J., and Subhash, N. (2014). Efficacy of antimicrobial photodynamic therapy in the management of chronic periodontitis: a randomized controlled clinical trial. J. Clin. Periodontol. 41, 573–581. doi: 10.1111/jcpe.12249
15	Carvalho et al.	2015	RCT	Study group: PDT Control group: Irrigation	Chronic periodontitis	Methylene blue	0.01%	Diode laser	–	660 nm	40 mW	90 J/cm^2^	90 s	4	3 months	*A. actinomycetemcomitans, P. gingivalis, Treponema denticola, Tannerella forsythia*	Baseline, 3, 6, 9, and 12 months	No significant differences were observed between the study and control groups.	Carvalho, V. F., Andrade, P. V. C., Rodrigues, M. F., Hirata, M. H., Hirata, R. D. C., Pannuti, C. M., et al. (2015). Antimicrobial photodynamic effect to treat residual pockets in periodontal patients: a randomized controlled clinical trial. J. Clin. Periodontol. 42, 440–447. doi: 10.1111/jcpe.12393
16	Müller Campanile et al.	2015	RCT	Group 1: Ultrasonic debridement + twice within 1 week PDT Group 2: Ultrasonic debridement + only once PDT Group 3: Ultrasonic debridement	Periodontitis (Patients Under maintenance phase)	Methylene blue	–	Diode laser	–	670 nm	280 mW	–	60 s	1–2	1 week	*Porphyromonas gingivalis, Aggregatibacter actinomycetemcomitans, Tannerella forsythia, Treponema denticola, Prevotella intermedia,* and *Parvimonas micra*	Baseline, 3 and 6 months	A single or double episodes of PDT had some additional benefit over ultrasonic instrumentation alone.	Müller Campanile, V. S., Giannopoulou, C., Campanile, G., Cancela, J. A., and Mombelli, A. (2015). Single or repeated antimicrobial photodynamic therapy as adjunct to ultrasonic debridement in residual periodontal pockets: clinical, microbiological, and local biological effects. Lasers Med. Sci. 30, 27–34. doi: 10.1007/s10103-013-1337-y
17	Castro Dos Santos et al.	2016	RCT	Study group: SRP + PDT Control group: SRP	Chronic periodontitis	Methylene blue	0.005%	Diode laser	Continuous mode	660 nm	2.15 W/cm^2^	129 J/cm^2^	60 s	1	–	–	1, 3, and 6 months	Between the two groups, none of the evaluated clinical parameters showed statistically significant differences.	Castro Dos Santos, N. C., Andere, N. M., Araujo, C. F., de Marco, A. C., Dos Santos, L. M., Jardini, M. A., et al. (2016). Local adjunct effect of antimicrobial photodynamic therapy for the treatment of chronic periodontitis in type 2 diabetics: split-mouth double-blind randomized controlled clinical trial. Lasers Med. Sci. 31, 1633–1640. doi: 10.1007/s10103-016-2030-8
18	Theodoro et al.	2017	RCT	Study group: SRP + PDT Control group: SRP + 400 mg metronidazole and 500 mg amoxicillin	Chronic periodontitis	Methylene blue	10 mg/ml	Diode laser	–	660 nm	100 mW	160 J/cm^2^	48 s	3	2 days	–	3 months	Both groups showed notable reductions in PPD, BOP, and CAL compared to baseline. PDT led to a significant reduction in CAL in the intermediate pocket for intergroup comparison.	Theodoro, L. H., Lopes, A. B., Nuernberg, M. A. A., Cláudio, M. M., Miessi, D. M. J., Alves, M. L. F., et al. (2017). Comparison of repeated applications of PDT with amoxicillin and metronidazole in the treatment of chronic periodontitis: a short-term study. J. Photochem. Photobiol. B 174, 364–369. doi: 10.1016/j.jphotobiol.2017.08.012
19	Bechara Andere et al.	2018	RCT	Study group 1: UPD + CLM Study group 2: UPD + PDT Study group 3: UPD + CLM + PDT Control group: UPD	Aggressive periodontitis	Methylene blue	10 mg/ml	Diode laser	–	660 nm	60 mW	129 J/cm^2^	60 s	1	–	–	3 and 6 months	Compared to the control group, all study groups demonstrated a reduction in PPD at 3 months. However, at 6 months, the reduction in PPD was greater in study group 1 and 3. Study group 3 showed a notable improvement in CAL compared to study group 2 and the control group.	Bechara Andere, N. M. R., Dos Santos, N. C. C., Araujo, C. F., Mathias, I. F., Rossato, A., de Marco, A. C., et al. (2018). Evaluation of the local effect of nonsurgical periodontal treatment with and without systemic antibiotic and photodynamic therapy in generalized aggressive periodontitis. A randomized clinical trial. Photodiagnosis Photodyn. Ther. 24, 115–120. doi: 10.1016/j.pdpdt.2018.09.002
20	Theodoro et al.	2018	RCT	Study group: SRP + PDT Control group: SRP + 400 mg metronidazole and 500 mg amoxicillin	Chronic periodontitis	Methylene blue	10 mg/ml	Diode laser	–	660 nm	100 mW	160 J/cm^2^	48 s	3	2 days	*Porphyromonas gingivalis, Prevotella nigrescens,* and *Prevotella intermedia*	3 and 6 months	Significant reductions in PPD, BOP, CAL, P. intermedia, and P. nigrescens were observed in both groups compared to baseline. Between-group comparisons were non- significant.	Theodoro, L. H., Assem, N. Z., Longo, M., Alves, M. L. F., Duque, C., Stipp, R. N., et al. (2018). Treatment of periodontitis in smokers with multiple sessions of antimicrobial photodynamic therapy or systemic antibiotics: a randomized clinical trial. Photodiagnosis Photodyn. Ther. 22, 217–222. doi: 10.1016/j.pdpdt.2018.04.003
21	Vohra et al.	2018	RCT	Study group: SRP + PDT Control group: SRP	Chronic periodontitis	Methylene blue	0.005%	Diode laser	–	670 nm	150 mW	–	–	1	–	–	6 and 12 weeks	Significant reduction in PPD of 4–6 mm and ≥ 7 mm was observed for PDT group as compared to the SRP group at both 6 weeks and 12 weeks. Inter-group comparison showed significant difference in TNF-α and IL-6 levels for PDT group at 12 weeks.	Vohra, F., Akram, Z., Bukhari, I. A., Sheikh, S. A., and Javed, F. (2018). Short-term effects of adjunctive antimicrobial photodynamic therapy in obese patients with chronic periodontitis: a randomized controlled clinical trial. Photodiagnosis Photodyn. Ther. 21, 10–15. doi: 10.1111/j.1600-051X.2008.01303.x
22	Alvarenga et al.	2019	RCT	Group 1: 1 min PDT + surfactant Group 2: 3 min PDT + surfactant Group 3: 5 min PDT + surfactant Group 4: 1 min PDT Group 5: 3 min PDT Group 6: 5 min PDT	Chronic periodontitis	Methylene blue	1 μM	Red laser	–	660 nm	250 mW/cm^2^	15, 45 and 75 J/cm^2^	1, 3, 5 min	1	–	–	Immediately after irradiation	Methylene blue in the surfactant vehicle produced microbial reduction in the group irradiated for 5 min. Spectroscopy showed that surfactant vehicle decreased the dimer peak signal at 610 nm.	Alvarenga, L. H., Gomes, A. C., Carribeiro, P., Godoy-Miranda, B., Noschese, G., Simões Ribeiro, M., et al. (2019). Parameters for antimicrobial photodynamic therapy on periodontal pocket—randomized clinical trial. Photodiagnosis Photodyn. Ther. 27, 132–136. doi: 10.1016/j.pdpdt.2019.05.035
23	Katsikanis et al.	2020	RCT	Study group: SRP + PDT Control group: SRP	Chronic periodontitis	Methylene blue	1%	GaAlAs diode laser	–	670 nm	445 mW/cm^2^	–	60 s	3	1 week	–	3 and 6 months	All groups lead to statistically significant improvements in BOP, CAL, PPD, and PI at 3 months and 6 months compared with baseline. There was no statistically significant difference regarding PD and BOP between groups.	Katsikanis, F., Strakas, D., and Vouros, I. (2020). The application of antimicrobial photodynamic therapy (aPDT, 670 nm) and diode laser (940 nm) as adjunctive approach in the conventional cause-related treatment of chronic periodontal disease: a randomized controlled split-mouth clinical trial. Clin. Oral Investig. 24, 1821–1827. doi: 10.1007/s00784-019-03045-1
24	Cláudio et al.	2021	RCT	Study group: SRP + PDT Control group: SRP	Chronic periodontitis	Methylene blue	10 mg/ml	Diode laser	–	660 nm	100 mW	157 J/cm^2^	50 s	1	–	*Porphyromonas gingivalis* and *Prevotella intermedia*	3 and 6 months	Both groups showed reduction in PPD at 90 and 180 days. The SRP + PDT group exhibited reduced PD means in deep pockets at 180 days. No differences were observed in *P. gingivalis* and *P. intermedia* levels.	Cláudio, M. M., Nuernberg, M. A. A., Rodrigues, J. V. S., Belizário, L. C. G., Batista, J. A., Duque, C., et al. (2021). Effects of multiple sessions of antimicrobial photodynamic therapy (aPDT) in the treatment of periodontitis in patients with uncompensated type 2 diabetes: a randomized controlled clinical study. Photodiagnosis Photodyn. Ther. 35:102451. doi: 10.1016/j.pdpdt.2021.102451
25	ALHarthi et al.	2022	RCT	Study group: MI + PDT Control group: MI	Chronic periodontitis and peri implant diseases (patients with or without depression)	Methylene blue	1%	Gallium Aluminium Arsenide laser	–	670 nm	350 or 440 mW/cm^2^	–	60 s lingually/palatally and 60 s buccally	–	–	–	4 months	In healthy patients, PDT offers no additional benefits in the treatment of periodontal inflammation.	ALHarthi, S. S., Divakar, D. D., Alwahibi, A., & BinShabaib, M. S. (2022). Effect of mechanical instrumentation with adjunct photodynamic therapy on salivary TNFα levels and clinical periodontal and peri-implant status in patients with depression: A randomized controlled trial. Photodiagnosis and photodynamic therapy, 40, 103042. doi: 10.1016/j.pdpdt.2022.103042
26	Andere et al.	2022	RCT	Group 1: PDT Group 2: OFD	Chronic periodontitis	Methylene blue	10 mg/ml	Diode laser	–	660 nm	60 mW	129 J/cm^2^	60 s	5	1, 5, 7 days	*A.actinomycetemcomitans* and *P. gingivalis*	Baseline, 3, 6, and 12 months	Compared to PDT, OFD was superior in reducing PPD in deep pockets. However, OFD resulted in greater gingival recession. Both treatments lowered *P. gingivalis* levels but only OFD reduced levels of *A. actinomycemtemcomitans*.	Andere, N. M. R. B., Castro Dos Santos, N. C., Araújo, C. F., Paz, H. E. S., Shaddox, L. M., Casarin, R. C. V., & Santamaria, M. P. (2022). Open flap debridement compared to repeated applications of photodynamic therapy in the treatment of residual pockets: A randomized clinical trial. Journal of periodontology, 93(11), 1671–1681. doi: 10.1002/JPER.22-0059
27	Elsadek et al.	2022	RCT	Study group: SRP + PDT Control group: SRP	Chronic necrotizing ulcerative periodontitis	Methylene blue	0.005%	Diode laser	–	660 nm	140 mW	300 J/cm^2^	40–45 s	1	–	*Aggregatibacter actinomycetemcomitans, Porphyromonas gingivalis* and *Tannerella forsythia*	Baseline, 3 and 6 months	PDT as a supplement to SRP resulted in improved clinical periodontal outcomes and a reduction in bacterial content for patients with necrotizing ulcerative periodontitis.	Elsadek M. F. (2022). Clinical and bacterial outcomes of photodynamic therapy in the treatment of chronic necrotizing ulcerative periodontitis. Photodiagnosis and photodynamic therapy, 39, 102977. doi: 10.1016/j.pdpdt.2022.102977
28	Elsadek et al.	2022	RCT	Study group: SRP + PDT Control group: SRP	Chronic periodontitis	Methylene blue	0.005%	Diode laser	–	670 nm	1.1 W/cm^2^	–	–	1	–	–	Baseline, 3 and 6 months	PDT contributed to reduce the clinical and pro-inflammatory load within the diseased periodontal pockets in geriatric patients.	Elsadek, M. F., & Farahat, M. F. (2022). Effectiveness of photodynamic therapy as an adjunct to periodontal scaling for treating periodontitis in geriatric patients. European review for medical and pharmacological sciences, 26(6), 1832–1838. doi: 10.26355/eurrev_202203_28327
29	Soundarajan and Rajasekar	2022	RCT	Study group 1: SRP + Er, Cr: YSGG laser Study group 2: SRP + PDT Control group: SRP	Chronic periodontitis	Methylene blue	1%	Diode laser	Continuous wave mode	660 nm	28 mW/cm^2^	16.72 J/cm^2^	10 s	4	1 week	–	3 months	PI, GI PD, and CAL significantly improved at 3 months follow up compared to baseline in both the study groups. Adjunctive use of Er, Cr:YSGG laser showed better clinical improved clinical outcomes compared with PDT + SRP and control.	Soundarajan, S., and Rajasekar, A. (2022). Comparative evaluation of combined efficacy of methylene blue mediated antimicrobial photodynamic therapy (a-PDT) using 660 nm diode laser versus erbium-chromium-yttrium-scandium-gallium-garnet (Er, Cr: YSGG) laser as an adjunct to scaling and root planing on clinical parameters in supportive periodontal therapy: a randomized split-mouth trial. Photodiagnosis Photodyn. Ther. 39:102971. doi: 10.1016/j.pdpdt.2022.102971
30	Coelho et al.	2023	RCT	Study group: SRP + PDT Control group: SRP	Chronic periodontitis	Methylene blue	1%	Diode laser	–	670 nm	0.25 mW/cm^2^	2.49 J/cm^2^ (per site); 14.94 J/cm^2^ (per tooth)	10 s	2	1 week	–	Baseline and 3 months	Both groups showed progress in clinical parameters. In the test group, there were greater decreases in probing depth and bleeding on probing. There were no significant differences in the other clinical parameters between groups.	Coelho, T. D. R. C., Pinto Filho, J. M., Ribeiro Caponi, L. S. F. E., Soares, J. D. M., Dos Santos, J. N., & Cury, P. R. (2023). Photodynamic therapy as an adjunctive treatment for Grade C periodontitis in molar teeth: a preliminary trial. Quintessence international (Berlin, Germany: 1985), 54(7), 528–534. doi: 10.3290/j.qi.b3957661
31	Elsadek et al.	2023	RCT	Group 1: PGA/MB/AV + SRP Group 2: PDT + SRP Group 3: SRP	Chronic periodontitis	Methylene blue	0.005%	Diode laser	–	670 nm	1.1 W/cm^2^	–	60 s	3	1 week	*Tannerella forsythia* and *Porphyromonas gingivalis*	Baseline, 3 and 6 months	All three groups showed notable improvements in plaque scores and BOP at the 3-month and 6-month follow-ups. Group 2 showed a notable decrease in BOP scores compared to the other groups. Both microbial species exhibited a statistically significant reduction in Group 2 after 3 months of follow-up.	Elsadek MF, Almoajel A. Clinical and bacterial periodontal parameters with methylene blue-loaded nanoparticles incorporated in a natural plant-based vehicle for the treatment of Stage III Grade B periodontitis. Photodiagnosis and Photodynamic Therapy. 2023;42.
32	Kassa et al.	2023	RCT	Study group 1: PDT_MB_SDS Study group 2: PDT_MB Control group 1: CTR_MB Control group 2: CTR_MB_SDS	Chronic periodontitis	Methylene blue	100 μM	Diode laser	Continuous wave mode	660 nm	0.25 W/cm^2^	30 J/cm^2^	2 min	1	–	Total bacteria count	Baseline and 30 days	The effect of methylene blue in surfactant did not cause enough phototoxic effects that could promote reduction of PPD.	Kassa, C. T., Salviatto, L. T. C., Tortamano, A. C. A. C., Rost-Lima, K. S., Damante, C. A., Pavani, C., Deana, A., Kato, I. T., Wainwright, M., & Prates, R. A. (2023). Antimicrobial photodynamic therapy mediated by methylene blue in surfactant vehicle as adjuvant to periodontal treatment. Randomized, controlled, double-blind clinical trial. Photodiagnosis and photodynamic therapy, 41, 103194. doi: 10.1016/j.pdpdt.2022.103194
33	Rodrigues et al.	2023	RCT	Study group: SRP + PDT Control group: SRP + sham PDT	Chronic periodontitis	Methylene blue	1%	Diode laser	–	660 nm	0.25 mW/cm^2^	2.49 J/cm^2^ (per site); 14.94 J/cm^2^ (per tooth)	10 s	2	1 week	–	Every 3 weeks for 90 days	Using PDT in conjunction with SRP led to slightly superior periodontal clinical results than using SRP alone, exerting a superior effect at sites with greater baseline PPD.	Rodrigues, R. D., Araujo, N. S., Filho, J. M. P., Vieira, C. L. Z., Ribeiro, D. A., Dos Santos, J. N., & Cury, P. R. (2023). Photodynamic therapy as adjunctive treatment of single-rooted teeth in patients with grade C periodontitis: A randomized controlled clinical trial. Photodiagnosis and photodynamic therapy, 44, 103776. doi: 10.1016/j.pdpdt.2023.103776
34	Shetty et al.	2023	RCT	Study group: NSPT + PDT Control group: NSPT	Chronic periodontitis (prediabetic and non-diabetic patients)	Methylene blue	100 μM	Diode laser	–	670 nm	150 mW	–	–	1	–	–	Baseline and 3 weeks	A single session of NSPT, whether combined with PDT or not, decreases periodontal inflammation but does not influence glycemic levels in prediabetic patients.	Shetty, B., Divakar, D. D., Jameel, A. H. A., Almalki, S. A., Gowdar, I. M., & Dewan, H. (2023). Effect of non-surgical periodontal therapy with adjunct photodynamic therapy on periodontal and glycemic statuses in prediabetic patients with periodontal disease. Photodiagnosis and photodynamic therapy, 42, 103362. doi: 10.1016/j.pdpdt.2023.103362
35	Cláudio et al.	2024	RCT	Group 1: SI Group 2: SI + Blue®m (BM) formula Group 3: SI + Blue®m (BM) formula + PDT	Chronic periodontitis (type 2 diabetic patients)	Methylene blue	100 μg/ml	Diode laser	–	660 ± 10 nm	100 mW	166 J/cm^2^	50 s	1	–	–	90 and 180 days	For patients with poorly controlled type 2 diabetes mellitus, the application of an adjuvant active oxygen-releasing gel, either with or without PDT, led to the same clinical benefits in the treatment of residual pockets.	Cláudio, M. M., Garcia, V. G., Freitas, R. M., Rodrigues, J. V. S., Wainwright, M., Casarin, R. C. V., Duque, C., & Theodoro, L. H. (2024). Association of active oxygen-releasing gel and photodynamic therapy in the treatment of residual periodontal pockets in type 2 diabetic patients: A randomized controlled clinical study. Journal of periodontology, 95(4), 360–371. doi: 10.1002/JPER.23-0125
36	Cunha et al.	2024	RCT	Study group 1: T1D patients + SRP Study group 2: T1D patients + SRP + PDT Control group 1: Normoglycemic patients + SRP Control group 2: Normoglycemic patients + SRP + PDT	Chronic periodontitis	Methylene blue	10 mg/ml	Diode laser	–	650 ± 10 nm	3.57 W/cm^2^	285.7 J/cm^2^	80 s	3	1 week	–	Baseline, 1, 3 and 6 months	The use of adjuvant PDT resulted in improved periodontal clinical measures and a reduction in inflammatory cytokines in both T1D and normoglycemic individuals. Nonetheless, patients with normal blood sugar levels experienced more significant clinical improvements than those with T1D following adjuvant PDT treatment.	Cunha, P. O., Gonsales, I. R., Greghi, S. L. A., Sant'ana, A. C. P., Honório, H. M., Negrato, C. A., Zangrando, M. S. R., & Damante, C. A. (2024). Adjuvant antimicrobial photodynamic therapy improves periodontal health and reduces inflammatory cytokines in patients with type 1 diabetes mellitus. Journal of applied oral science: revista FOB, 32, e20240258. doi: 10.1590/1678-7757-2024-0258
37	Nie et al.	2024	RCT	Study group 1: SRP + A single PDT Study group 2: SRP + Three repeated PDT Control group: SRP	Chronic periodontitis	Methylene blue	0.01%	Diode laser	–	650–670 nm	2 mW/cm^2^	–	60 s	1, 3	3 repeated PDT applications in 1 week	*Streptococcus, Actinomyces, Porphyromonas, Fusobacterium, Rothia, Lautropia, Neisseria, Treponema_2, Capnocytophaga, Leptotrichia, Haemophilus, Fretibacterium, Prevotella,* and *Veillonella*	Baseline and 8 weeks	PDT promotes alterations in the microbial makeup of subgingival plaque in periodontitis patients, steering it towards improved periodontal health.	Nie, M., Huang, P., Peng, P., Shen, D., Zhao, L., Jiang, D., Shen, Y., Wei, L., Bible, P. W., Yang, J., Wang, J., & Wu, Y. (2024). Efficacy of photodynamic therapy as an adjunct to scaling and root planing on clinical parameters and microbial composition in subgingival plaque of periodontitis patients: A split-mouth randomized clinical trial. Journal of periodontology, 95(6), 535–549. doi: 10.1002/JPER.23-0195
38	Najm et al.	2024	RCT	Study group 1: RSD + MB-PDT Study group 2: RSD + TBO-PDT Control group: RSD	Chronic periodontitis	Methylene blue; toluidine blue O	1 mg/ml	LED	–	635 nm	–	120 J/cm^2^	1 min	2	2 weeks	–	3 months	The adjunctive application of MB and TBO to RSD can notably enhance periodontal pocket closure and diminish inflammation signs. Additionally, TBO is seemingly more efficient in addressing deep periodontal pockets, while MB is better suited for shallower pockets.	Najm, K. K., Gul, S. S., & Abdulkareem, A. A. (2024). Efficacy of Non-Surgical Periodontal Therapy with Adjunctive Methylene Blue and Toluidine Blue O Mediated Photodynamic in Treatment of Periodontitis: A Randomized Clinical Trial. Clinics and practice, 14(3), 954–964. doi: 10.3390/clinpract14030076
39	Novaes Jr. et al.	2012	RCT	Study group: PDT Control group: SRP	Aggressive periodontitis	Phenothiazine chloride	–	Diode laser	–	660 nm	60 mW/cm^2^	–	10 s	1	–	*A. actinomycetemcomitans, T. forsythia,* and *P. gingivalis*	3 months	A single session of PDT and SRP influenced various bacterial groups. PDT proved to be more efficient in decreasing the counts of *A. actinomycetemcomitans*.	Novaes, A. B. Jr., Schwartz-Filho, H. O., de Oliveira, R. R., Feres, M., Sato, S., and Figueiredo, L. C. (2012). Antimicrobial photodynamic therapy in the non-surgical treatment of aggressive periodontitis: microbiological profile. Lasers Med. Sci. 27, 389–395. doi: 10.1007/s10103-011-0901-6
40	Arweiler et al.	2014	RCT	Study group: SRP + PDT Control group: SRP + 375 mg amoxicillin and 250 mg metronidazole	Aggressive periodontitis	Phenothiazine chloride	–	Diode laser	–	660 nm	–	–	60 s	2	–	–	3 months	In both groups, PPD, BOP, and CAL significantly decreased from the baseline. Compared to PDT, antibiotics led to significantly decrease in PD and a reduced number of pockets ≥7 mm.	Arweiler, N. B., Pietruska, M., Pietruski, J., Skurska, A., Dolińska, E., Heumann, C., et al. (2014). Six-month results following treatment of aggressive periodontitis with antimicrobial photodynamic therapy or amoxicillin and metronidazole. Clin. Oral Investig. 18, 2129–2135. doi: 10.1007/s00784-014-1193-6
41	Moreira et al.	2015	RCT	Study group: SRP + PDT Control group: SRP	Aggressive periodontitis	Phenothiazine chloride	10 mg/ml	Diode laser	–	670 nm	250 mW/cm^2^	2.49 J/cm^2^ (per site); 14.94 J/cm^2^ (per tooth)	10 s	4	2, 7 and 14 days	*Periodontal pathogens such as A. actinomycetemcomitans* and *species of orange and red complexes*	3 months	PDT significantly decreased PPD, CAL at 90 days. PDT also exhibited significantly less periodontal pathogens of red and orange complexes compared to control.	Moreira, A. L., Novaes, A. B. Jr., Grisi, M. F., Taba, M. Jr., Souza, S. L., Palioto, D. B., et al. (2015). Antimicrobial photodynamic therapy as an adjunct to non-surgical treatment of aggressive periodontitis: a split-mouth randomized controlled trial. J. Periodontol. 86, 376–386. doi: 10.1902/jop.2014.140392
42	Petelin et al.	2015	RCT	Group 1: Hand SRP Group 2: Ultrasonic SRP Group 3: Ultrasonic SRP + PDT	Chronic periodontitis	Phenothiazine chloride	–	Diode laser	–	660 nm	60 mW	–	60 s	3	2 or 4 days	*Aggregatibacter actinomycetemcomitans, P. gingivalis, Prevotella intermedia, Tannerella, forsythia,* and *Treponema denticola*	Baseline, 3, 6, 9, and 12 months	Adjunctive PDT was more effective than SRP alone in reducing BOP and microbial burden at the 3 and 12 months. PDT resulted in a greater reduction of periodontal pathogens compared to mechanical debridement alone.	Petelin, M., Perkič, K., Seme, K., and Gašpirc, B. (2015). Effect of repeated adjunctive antimicrobial photodynamic therapy on subgingival periodontal pathogens in the treatment of chronic periodontitis. Lasers Med. Sci. 30, 1647–1656. doi: 10.1007/s10103-014-1632-2
43	Queiroz et al.	2015	RCT	Study group: SRP + PDT Control group: SRP	Chronic periodontitis	Phenothiazine chloride	10 mg/ml	Diode laser	–	660 nm	28 mW/cm^2^	2.79 J/cm^2^ (per site); 16.72 J/cm^2^ (per tooth)	10 s	1	–	–	Baseline, 7, 30, and 90 days	No differences were observed in intragroup comparisons. The adjunctive effect of PDT did not warrant improvements on clinical parameters in smokers.	Queiroz, A. C., Suaid, F. A., de Andrade, P. F., Oliveira, F. S., Novaes, A. B., Taba, M., et al. (2015). Adjunctive effect of antimicrobial photodynamic therapy to nonsurgical periodontal treatment in smokers: a randomized clinical trial. Lasers Med. Sci. 30, 617–625. doi: 10.1007/s10103-013-1379-1
44	Martins et al.	2017	RCT	Study group: PDT + ST Control group: ST only	Chronic periodontitis	Phenothiazine chloride	10 mg/ml	Red laser	–	–	28 mW/cm^2^	2.79 J/cm^2^ (per site); 16.72 J/cm^2^ (per tooth)	10 s	1	–	40 bacterial species	2, 3, and 5 months	PDT presented a significantly higher decrease in PPD than the Control Group at 90 days. PDT Group also demonstrated significantly less periodontal pathogens of red complex (*Treponema denticola*).	Martins, S. H. L., Novaes, A. B. Jr., Taba, M. Jr., Palioto, D. B., Messora, M. R., Reino, D. M., et al. (2017). Effect of surgical periodontal treatment associated to antimicrobial photodynamic therapy on chronic periodontitis: a randomized controlled clinical trial. J. Clin. Periodontol. 44, 717–728. doi: 10.1111/jcpe.12744
45	Tabenski et al.	2017	RCT	Study group: SRP + PDT Control group: SRP + minocycline hydrochloride microspheres	Chronic periodontitis	Phenothiazine chloride	–	Diode laser	–	670 nm	75 mW/cm^2^	–	10 s	2	1 week	*A. actinomycetemcomitans, P. gingivalis, Tannerella forsythia (T.f.),* and *Treponema denticola (T.d.)*	6 weeks, 3, 6, and 12 months	Significant improvements in clinical and microbiological parameters were found in two groups compared to baseline. Between-group comparisons were not statistically significant.	Tabenski, L., Moder, D., Cieplik, F., Schenke, F., Hiller, K. A., Buchalla, W., et al. (2017). Antimicrobial photodynamic therapy vs. local minocycline in addition to non-surgical therapy of deep periodontal pockets: a controlled randomized clinical trial. Clin. Oral Investig. 21, 2253–2264. doi: 10.1007/s00784-016-2018-6
46	Cadore et al.	2019	RCT	Study group: PDT + ST Control group: ST	Chronic periodontitis	Phenothiazine chloride	10 mg/ml	Diode laser	–	660 nm	60 mW/cm^2^ (maximum power)	0.6 J/cm^2^	60 s	4	2, 5, 7 days	40 subgingival microbial species	2 and 5 months	A significant reduction in PD was observed at 150 days for the PDT, when compared with the control group. CAL gain was significantly higher in the test group at 60 and 150 days. Changes in the subgingival microbiota were similar between the groups, but the PDT revealed a larger number of bacteria associated with periodontal disease at 150 days compared to control.	Cadore, U. B., Reis, M. B. L., Martins, S. H. L., Invernici, M. M., Novaes, A. B. Jr., Taba, M. Jr., et al. (2019). Multiple sessions of antimicrobial photodynamic therapy associated with surgical periodontal treatment in patients with chronic periodontitis. J. Periodontol. 90, 339–349. doi: 10.1002/JPER.18-0373
47	de Melo Soares et al.	2019	RCT	Study group: SRP + PDT Control group: SRP	Chronic periodontitis	Phenothiazine chloride	10 mg/ml	Diode soft laser	–	660 nm	28 mW/cm^2^	2.79 J/cm^2^ (per site); 16.72 J/cm^2^ (per tooth)	10 s	4	2, 5, 7 days	40 bacterial species	Baseline, 14, 30, and 90 days	PDT combined with SRP did not result in statistically significant enhancements in clinical parameters over SRP alone.	de Melo Soares, M. S., D’Almeida Borges, C., de Mendonça Invernici, M., Frantz, F. G., de Figueiredo, L. C., de Souza, S. L. S., et al. (2019). Antimicrobial photodynamic therapy as adjunct to non-surgical periodontal treatment in smokers: a randomized clinical trial. Clin. Oral Investig. 23, 3173–3182. doi: 10.1007/s00784-018-2740-3
48	Mocanu et al.	2021	RCT	Study group 1: SRP + Chlorhexidine rinsing Study group 2: SRP + PDT Control group: SRP	Chronic periodontitis	Phenothiazine chloride	–	Laser light	–	660 nm	100 mW	–	10 s	3	1 week	*Aggregatibacter actinomycetemcomitans, Porphyromonas gingivalis, Tannerella forsythia,* and *Treponema denticola*	1 and 6 months	PDT led to notable enhancements in both clinical and microbiological burden after one month, and these improvements persisted for 6 months.	Mocanu, R. C., Martu, M.-A., Luchian, I., Sufaru, I. G., Maftei, G. A., Ioanid, N., et al. (2021). Microbiologic profiles of patients with dental prosthetic treatment and periodontitis before and after Photoactivation therapy—randomized clinical trial. Microorganisms 9:713. doi: 10.3390/microorganisms9040713
49	Arsic et al.	2022	RCT	Study group: SRP + PDT Control group: SRP	Chronic periodontitis	Phenothiazine chloride	–	Laser light	–	660 nm	100 mW	–	10 s	1	–	*Aggregatibacter actinomycetemcomitans, Porphyromonas gingivalis, and Treponema denticola*	7 days	PDT + SRP led to a statistically significant improvement in both clinical parameters and microbiological status compared to SRP alone.	Arsic, Z., Jovanovic, R., Djordjevic, A., Sehalic, M., Marjanovic, D., Mikic, M., et al. (2022). Clinical and microbiological effects of photodynamic therapy applied in non-surgical treatment of periodontitis. Vojnosanit. Pregl. 79, 17–24. doi: 10.2298/VSP200304060A
50	Alshibani et al.	2022	RCT	Study group: NSPT + PDT Control group: NSPT	Chronic periodontitis	Photosensitizer based on rhizome of ginger (0.005%)	–	Diode laser	–	660 nm	150 mW	–	60 s	–	–	–	3 months	All treatments resulted in significant statistical improvements PI, BI and PD at 3 months compared with their respective baseline, and there were no significant differences in the study and control groups.	Alshibani, N., Alssum, L., Basudan, A., Shaheen, M., Alqutub, M. N., Dahash, F. A., & Alkattan, R. (2022). Non-surgical periodontal therapy with adjunct photodynamic therapy for the management of periodontal inflammation in adults using nicotine-free electronic-cigarette: A randomized control trial. Photodiagnosis and photodynamic therapy, 38, 102820. doi: 10.1016/j.pdpdt.2022.102820
51	Nedumaran et al.	2024	RCT	Study group: SRP + PDT Control group: SRP	Chronic periodontitis	Rose bengal	–	Diode laser	Continuous mode	650 nm	200 mW	0.5–0.9 J/cm^2^ (per tooth)	10 s	1	–	*P. gingivalis, T. denticola,* and *Tannerella forsythia*	Baseline and 3 months	RB-PDT combined with SRP significantly improved GI, PPD, and CAL, and reduced microbial numbers more effectively than SRP alone in managing chronic periodontitis.	Nedumaran, N., & Rajasekar, A. (2024). Efficacy of Rose Bengal-Mediated Antimicrobial Photodynamic Therapy as an Adjunct to Scaling and Root Planing on Clinical and Microbiological Parameters in the Management of Chronic Periodontitis: A Single-Blinded, Randomized Controlled Clinical Trial. Photobiomodulation, photomedicine, and laser surgery, 42(8), 561–567. doi: 10.1089/pho.2024.0037
52	Cosgarea et al.	2021	RCT	Study group 1: SI + PDT Study group 2: SI + LDD Control group: SI	Chronic periodontitis	HELBO Blue	–	Laser light	–	660 nm	100 mW	–	30 s	2	1 week	*Aggregatibacter actinomycetemcomitans, Porphyromonas gingivalis, Tannerella forsythia, Prevotella intermedia, Treponema denticola, Fusobacterium nucleatum, Campylobacter rectus,* and *Filifactor allocis*	3 and 6 months	All three treatments had statistically significant improvements of all clinical parameters without significant differences between groups.	Cosgarea, R., Eick, S., Batori-Andronescu, I., Jepsen, S., Arweiler, N. B., Rößler, R., et al. (2021). Clinical and microbiological evaluation of local doxycycline and antimicrobial photodynamic therapy during supportive periodontal therapy: a randomized clinical trial. Antibiotics 10:277. doi: 10.3390/antibiotics10030277
53	Cosgarea et al.	2022	RCT	Group 1: SI + PDT Group 2: SI + LDD Control group: SI	Chronic periodontitis	HELBO Blue	–	Laser light	–	660 nm	100 mW	–	10 s	2	1 week	*A. actinomycetemcomitans, P. gingivalis, T. forsythia, T. denticola, Parvimonas micra, F. nucleatum, Camphilobacter,* and *Filifactor allocis*	12 months	All treatments resulted in significant statistical improvements in clinical parameters, and there were no significant differences among the groups.	Cosgarea, R., Ramseier, C. A., Jepsen, S., Arweiler, N. B., Jervøe-Storm, P. M., Batori-Andronescu, I., et al. (2022). One-year clinical, microbiological and immunological results of local doxycycline or antimicrobial photodynamic therapy for recurrent/persisting periodontal pockets: a randomized clinical trial. Antibiotics11:738. doi: 10.3390/antibiotics11060738
54	Husejnagic et al.	2019	RCT	Study group: SRP + PDT Control group: SRP	Chronic periodontitis	Tolonium chloride	12.7 μg/ ml	LED	–	635 nm	750 mW (maximum output power)	14 J/cm^2^ (maximum energy density)	60 s	2	–	11 periopathogenic bacteria	Baseline, 12 weeks	No significant difference was seen in terms of clinical parameters between the control and study group.	Husejnagic, S., Lettner, S., Laky, M., Georgopoulos, A., Moritz, A., and Rausch-Fan, X. (2019). Photoactivated disinfection in periodontal treatment: a randomized controlled clinical split-mouth trial. J. Periodontol. 90, 1260–1269. doi: 10.1002/JPER.18-0576
55	Grzech-Leśniak et al.	2019	RCT	Study group: SRP + PDT Control group: SRP	Chronic periodontitis	Toluidine blue	0.1%	Diode laser	Continuous wave mode	635 nm	200 mW	117.64 J/cm^2^	60 s	3	1 week	8 bacterial species	Baseline, 3 and 6 months	The PDT group significantly decreased inflammation, BOP, and microbial load (except *A. actinomycetemcomitans*) compared to the control group.	Grzech-Leśniak, K., Gaspirc, B., and Sculean, A. (2019). Clinical and microbiological effects of multiple applications of antibacterial photodynamic therapy in periodontal maintenance patients. A randomized controlled clinical study. Photodiagnosis Photodyn. Ther. 27, 44–50. doi: 10.1016/j.pdpdt.2019.05.028
56	Mongardini et al.	2014	RCT	Study group: SRP + PDT Control group: SRP	Chronic periodontitis	Toluidine blue O	0.1 mg/ml	Diode laser	–	628 nm	2,000 mW/cm^2^	20 J/cm^2^	10 s	1	–	*Aggregatibacter actinomycetemcomitan, Porphyromonas gingivalis, Treponema denticola, Tannerella forsythia, Fusobacterium nucleatum spp.,* and *Prevotella intermedia*	Baseline, 1 week	The study group showed larger decreases in the number of microorganisms.	Mongardini, C., Di Tanna, G. L., and Pilloni, A. (2014). Light-activated disinfection using a light-emitting diode lamp in the red spectrum: clinical and microbiological short-term findings on periodontitis patients in maintenance. A randomized controlled split-mouth clinical trial. Lasers Med. Sci. 29, 1–8. doi: 10.1007/s10103-012-1225-x
57	Wang et al.	2024	RCT	Study group: SRP + PDT Control group: SRP	Chronic periodontitis	Toluidine blue O	0.01%	LED	–	660 nm	100 mW	–	1 min	2	1 month	–	Baseline, 1, 3 and 6 months	At 3 months, the BOP% and PI in the study were significantly lower than those in the control group. The study group showed a much larger increase in BOP% and PI than the control group three months following treatment.	Wang, X., W. Tang, Y. Jiang, Y. Shi, Z. Yan and D. Wang (2024). “Clinical observation of antibacterial photodynamic therapy assisted subgingival curettage for the treatment of chronic periodontitis.” Journal of Prevention and Treatment for Stomatological Diseases 32(6): 451–456

BOP, bleeding on probing; CAL, clinical attachment loss; CLM, clarithromycin; cm^2^, square centimeter; FMBS, full-mouth bleeding score; FMUD, full-mouth ultrasonic subgingival debridement; GI, gingival index; GB, gingival bleeding; LDD, local drug delivery; MB, Methylene blue; MI, mechanical instrumentation; mSBI, modified sulcular bleeding index; min, minutes; mW, milliwatts; nm, nanometers; NSPT, non-surgical periodontal therapy; OFD, open flap debridement; PGA/MB/AV, poly L glycolic acid nanoparticles-loaded methylene blue in aloe vera gel; PI, plaque index; PPD, pocket probing depth; RAL, relative attachment loss; RB, rose bengal; RCT, randomized clinical trial; RSD, root surface debridement; s, seconds; SBI, sulcus bleeding index; SDS, Sodium dodecyl sulfate; SI, subgingival instrumentation; SP gel, salvadora persica gel; ST, surgical periodontal treatment; SRP, scaling and root planning; T1D, type 1 diabetes; TBO, toluidine blue O; UPD, ultrasonic periodontal debridement; –, not reported.

In order to evaluate the clinical and radiographic outcomes of periodontitis patients treated with conventional mechanical debridement along with those treated with methylene blue-mediated adjunctive photodynamic treatment (MB-PDT), Alasqah et al. (33) conducted a meta-analysis of randomized controlled trials. The findings showed that when MB-PDT was used in conjunction with MD, as opposed to only MD, periodontal plaque index (PI), probing depth (PD), as well as bleeding on probing (BOP) scores exhibited significant improvements. Nevertheless, there was no statistically significant disparity in clinical attachment level (CAL) values seen between the control group (treated with MD alone) and the experimental group (treated with supplementary MB-PDT). The long-term effects of PDT and antibiotic therapy (amoxicillin 500 mg and metronidazole 400 mg for seven days) combined with traditional nonsurgical therapy in individuals with advanced periodontitis were compared in another investigation (34), proving that PDT and antibiotic therapy significantly improve clinical parameters (PD, CAL, and BOP) three months after treatment, and that stays also decreased six, nine, and twelve months later.

Ivanaga et al. (35) compared the clinical effectiveness of PDT with 100 mg/L curcumin (CUR) solution and LED irradiation (465–485 nm, 100 mW/cm^2^, 60 s) in treating residual pockets in type 2 diabetics, indicating that PDT group (SRP, irrigation with CUR solution and LED irradiation) showed a notable CAL gain at three months in comparison to baseline data compared with SRP group, CUR group (SRP and irrigation with CUR solution), and LED group (SRP and LED irradiation). All treatment groups showed reductions in PD and BOP in the intragroup comparison at three and six months. The result showed that the addition of PDT to SRP may yield short-term benefits in CAL gain among the type 2 diabetics with residual pockets.

*In vitro* experiments, Al-Ahmad et al. (36) tested the antimicrobial effects of PDT against a variety of periodontal pathogens, including subgingival dental biofilm and *Eikenella corrodens, Actinomyces odontolyticus, A. actinomycetemcomitans, Fusobacterium nucleatum, P. gingivalis, Atopobium rimae, Slackia exigua* and *Parvimonas micra*. It is showed that all tested periodontal pathogens and the PDT-treated subgingival biofilm were eliminated over the ranges of 3.43–8.34 and 3.91–4.28 log_10_ colony forming units (CFU), respectively.

## 5 Dental caries

Tooth caries is a multi-factorial, non-transmissible, biofilm-mediated disease that affects sensitive tooth hard tissues and is characterized by a phasic demineralization and remineralization phase. Dental caries is mostly caused by *Lactobacilli* and *Streptococcus mutans*, which produce acidity from carbohydrate metabolism, leading to an acidic pH and enamel demineralization (37). Dental caries has historically been frequently treated with fillings, eliminating carious tissue and cariogenic germs. However, insufficient clearance leads to a significant recurrence risk (secondary caries). PDT prevents cariogenic bacterial biofilm growth by damaging bacterial structure and decreasing acid production. Therefore, PDT is regarded as a successful adjuvant therapy for dental caries.

PDT can prevent dental caries by reducing microbial colonization and removing biofilm from the enamel surface. Alsaif et al. (38) studied the effects of erythrosine-PDT on *in vivo*-formed dental plaque biofilms using dental plaque samples from 18 healthy adult participants wearing intraoral appliances, showing that 220 M erythrosine-B (2 min or 15 min incubation times) with green light source (500–550 nm, 22.7 mW/cm^2^, 15 min continuous light or the fractionated light with 30 s light pulses ×5) reduced total bacterial counts (93%–95%) of *in vivo*-formed biofilms. Faria et al. (39) conducted a randomized controlled experiment to assess the effectiveness of composite restorations after selective caries removal (SCR) coupled with PDT. Following 12 months of observation, the group that had PDT treatment demonstrated a notably superior level of marginal adaption of the restoration in comparison to the control group.

Nie et al. (40) demonstrated that photodynamic inactivation based on Ce6 or methylene blue could be useful for preventing caries by controlling biofilms. Additionally, Ce6-mediated PDT produces more bactericidal activity (with an excess of 3 log_10_) than MB-PDT at the same PS concentration and light dose. Furthermore, PDT could impact the virulence characteristics of *Streptococcus mutans* as well (41); however, applying PDT to caries has only been studied in limited clinical trials, necessitating further *in vivo* proof to be considered valid (Table 4).

**Table 4:** Summary of studies on PDT for the treatment of dental caries.

	Study	Year	Study design	Treatment groups	Lesion type	Type of photosensitizers	Photosensitizers dose	Light source	Irradiation mode	Wave length	Fluence rate/Power	Energy density	Irradiation time	Treatment sessions	Treatment interval	Microorganisms	Follow up	Primary outcome	Referrence
1	Tahmassebi et al.	2015	RCT	Group 1: Control + PDT (varied concentrations of PS) Group 2: Control + PDT (varied light dose)	–	Erythrosine	22 and 220 μm	Tungsten filament Lamp	–	535 nm	22.7 mW/cm^2^	–	–	1	–	–	2 weeks	The erythrosine dose of 220 μM caused the most cell killing relative to controls. The most effective bactericidal methods were 15 min of continuous light exposure and light fractionation consisting of 5 × 1 min irradiations with 2 min dark recovery intervals.	Tahmassebi, J. F., Drogkari, E., and Wood, S. R. (2015). A study of the control of oral plaque biofilms via antibacterial photodynamic therapy. Eur. Arch. Paediatr. Dent. 16, 433–440. doi: 10.1007/s40368-014-0165-5
2	Alsaif et al.	2021	RCT	Control group 1: no Erythrosine, no light Control group 2: Erythrosine, no light Study group 1: PDT (continuous light) Study group 2: PDT (pulsed light)	–	Erythrosine	220 μm	Tungsten filament Lamp	Continuous mode or Pulse mode: 30 s light pulses (×5)	500–550 nm	22.7 mW/cm^2^	–	15 min or 5*30 s	1	–	–	2 weeks	Compared with the control group, the CFU in the study groups decreased significantly. There was no statistically significant difference between the four groups. Using either 2 min or 15 min incubation times and applying 15 min continuous irradiation showed significant reductions in total bacterial counts (∼ 93–95%) of in vivo-formed bio films.	Alsaif, A., Tahmassebi, J. F., and Wood, S. R. (2021). Treatment of dental plaque biofilms using photodynamic therapy: a randomised controlled study. Eur. Arch. Paediatr. Dent. 22, 791–800. doi: 10.1007/s40368-021-00637-y
3	Alves et al.	2019	RCT	Study group: PDT Control group: without PDT	Active caries and dentin cavitation	Methylene blue	0.005%	Diode laser	–	660 nm	100 mW	640 J/cm^2^	180 s	1	–	*Streptococcus mutans*	6 months	The reduction of s. Mutans CFU was 76.4% after caries removal, but associated with PDT it was 92.6%. After 6 months of clinical evaluation, it was found that there was no difference in retention, marginal adaptability, color, marginal discoloration and secondary dental caries between the groups.	Alves, L. V. G. L., Curylofo-Zotti, F. A., Borsatto, M. C., Salvador, S. L. S., Valério, R. A., Souza-Gabriel, A. E., & Corona, S. A. M. (2019). Influence of antimicrobial photodynamic therapy in carious lesion. Randomized split-mouth clinical trial in primary molars. Photodiagnosis and photodynamic therapy, 26, 124–130. doi: 10.1016/j.pdpdt.2019.02.018
4	Lima et al.	2022	RCT	Group 1: Biofilm before PDT Group 2: Biofilm 1 min after PDT Group 3: Biofilm before PDT Group 4: Biofilm 5 min after PDT	–	Methylene blue	0.01%	Diode laser	–	660 nm	–	90 J/cm^2^	100 s	1	–	–	–	Both groups exhibited a decrease in the number of bacteria. Group 4 showed the most significant reduction in bacteria.	Lima, N. G., Monteiro, R. M., Torres, C. P., de Souza-Gabriel, A. E., Watanabe, E., and Borsatto, M. C. (2022). Influence of antimicrobial photodynamic therapy with different pre-irradiation times on children’s dental biofilm: Randomized clinical trial. Eur. Arch. Paediatr. Dent. 23, 897–904. doi: 10.1007/s40368-022-00716-8
5	Ichinose Tsuno et al.	2014	RCT	Study group: PDT Control group: without PDT	–	Toluidine blue O	100, 500, and 1,000 μg/ml	LED	–	600–700 nm	1.1 W/cm^2^	–	20 s	6	–	–	4 days	PDT with 1,000 μg/ml TBO and red LED light significantly suppressed dental plaque formation without harming teeth or the surrounding tissues.	Ichinose-Tsuno, A., Aoki, A., Takeuchi, Y., Kirikae, T., Shimbo, T., Lee, M.-C.-I., et al. (2014). Antimicrobial photodynamic therapy suppresses dental plaque formation in healthy adults: a randomized controlled clinical trial. BMC Oral Health 14:152. doi: 10.1186/1472-6831-14-152
6	Melo et al.	2015	RCT	Study group: PDT Control group: without PDT	Deep caries lesions	Toluidine blue O	100 μg/ml	LED	–	630 nm	150 mW	94 J/cm^2^	–	1	–	*Streptococcus mutans* and *Lactobacillus spp.*	–	PDT resulted in a significant reduction in *mutans streptococci*, *Lactobacillus spp*. and total viable bacteria compared to the control.	Melo, M. A. S., Rolim, J. P. M. L., Passos, V. F., Lima, R. A., Zanin, I. C. J., Codes, B. M., et al. (2015). Photodynamic antimicrobial chemotherapy and ultraconservative caries removal linked for management of deep caries lesions. Photodiagnosis Photodyn. Ther. 12, 581–586. doi: 10.1016/j.pdpdt.2015.09.005
7	Steiner-Oliveira et al.	2015	RCT	Study group 1: TBO-PDT Study group 2: MB-PDT Control group: Chlorhexidine + RMGIC	Deciduous carious dentin	Toluidine blue O	0.1 mg/ml	LED	–	630 nm	100 mW	30 J/cm^2^	60 s	1	–	*Streptococcus mutans, Streptococcus sobrinus, Lactobacillus casei, Fusobacterium nucleatum* and *Atopobium rimae*	6 and 12 months	All groups were effective in reducing the number of microorganisms, except for *S. sobrinus*. There were no statistical differences noted between the protocols used.	Steiner-Oliveira, C., Longo, P. L., Aranha, A. C. C., Ramalho, K. M., Mayer, M. P. A., and de Paula Eduardo, C. (2015). Randomized in vivo evaluation of photodynamic antimicrobial chemotherapy on deciduous carious dentin. J. Biomed. Opt. 20:108003. doi: 10.1117/1.JBO.20.10.108003
						Methylene blue	0.01%	Red low-power laser	–	660 nm	100 mW	320 J/cm^2^	90 s	1	–				
8	Martins et al.	2023	RCT	Group 1: Caries removal with a low-speed drill (control group) Group 2: Partial Caries Removal + Papacarie™ Group 3: Partial Caries Removal + Papacarie™+Bixa orellana extract (20%) Group 4: Partial Caries Removal + Papacarie™ + Bixa orellana extract (20%) + LED	Deep caries lesions	Bixa orellana extract	20%	LED	–	440–480 nm	–	–	–	1	–	*Streptococcus*, and *lactobacilli*	Immediately, 1 week, and 1, 3, 6, and 12 months	–	Martins, L. F. B., de Sena, L. R., de Paula, D. M., Feitosa, V. P., Horliana, A. C. R. T., Fernandes, K. P. S., Mesquita-Ferrari, R. A., Motta, L. J., Gonçalves, M. L. L., & Bussadori, S. K. (2023). Investigation on the effect of antimicrobial photodynamic therapy as an adjunct for management of deep caries lesions-study protocol for a randomized, parallel groups, controlled clinical trial. Trials, 24(1), 165. doi: 10.1186/s13063-023-07181-8

cm^2^, square centimeter; min, minutes; mW, milliwatts; MB, methylene blue; nm, nanometers; μm, micrometer; RCT, randomized clinical trial; RMGIC, resin-modified glass ionomer cement; s, seconds; TBO, toluidine blue O; –, not reported.

## 6 Peri-implant diseases

Dental implants are crucial components of oral rehabilitation to restore missing teeth and enhance the quality of life of those with such therapeutic needs. Depending on the degree of the peri-implant tissue inflammatory pathological state, peri-implantitis can be subdivided into peri-implant mucositis and peri-implantitis. While peri-implantitis is an inflammatory condition resulting in cracked and absorbed alveolar bone, loosening of implants, and other risks, peri-implant mucositis is an inflammation of the mucous membrane around the implant (12). This element is a significant contributor to implant failure. The etiopathogenesis of peri-implant inflammatory disorders is related to *A. Actinomycetemcomitans* and *Treponema denticola* (*T. denticola*) (42). These microorganisms increase probing depth, plaque index, and gingival index (GI) surrounding implants, promoting crestal bone loss (CBL) as well as soft tissue inflammation (43, 44). The primary objective of peri-implantitis treatment is to eradicate the deleterious constituents of bacterial plaque in the vicinity of the implant. In most cases, peri-implantitis is treated with mechanical debridement using ultrasonic scalers, air-powered abrasives, and polishing brushes. However, these techniques have not entirely eradicated or effective inactivated peri-implant infections. This is primarily attributed to the complex nature of implant surfaces, which possess rough and microporous characteristics on macro- and microscopic scales (45). PDT has demonstrated successful infection cures from *Staphylococcus aureus*, *Pseudomonas aeruginosa*, *P. gingivalis*, and multidrug-resistant bacteria (46). And PDT, used with surgery, is more effective while preventing medication resistance and harm to nearby tissues (Table 5).

**Table 5:** Summary of studies on PDT for the treatment of peri-implant diseases.

	Study	Year	Study design	Treatment groups	Lesion type	Type of photosensitizers	Photosensitizers dose	Light source	Irradiation mode	Wave length	Fluence rate/Power	Energy density	Irradiation time	Treatment sessions	Treatment interval	Microorganisms	Follow up	Primary outcome	Referrence
1	Labban et al.	2021	RCT	Study group: PIMD + PDT Control group: PIMD	Peri-implantitis (DM2 patients)	Indocyanine green	1 mg/ml	Diode laser	Continuous mode	810 nm	200 mW	4 J	30 s (papilla); 10 s (pocket depth)	4	7 or 10 days	*Porphyromonas gingivalis* and *Treponema denticola*	Baseline, 3 and 6 months	In the treatment of type 2 diabetic patients with periimplantitis, the repeated application of indocyanine green mediated photodynamic therapy led to the improvement of clinical parameters (PPD, BOP, suppuration and PCBL) and microbial load.	Labban, N., Shibani, N. A., Al-Kattan, R., Alfouzan, A. F., Binrayes, A., & Assery, M. K. (2021). Clinical, bacterial, and inflammatory outcomes of indocyanine green-mediated photodynamic therapy for treating periimplantitis among diabetic patients: A randomized controlled clinical trial. Photodiagnosis and photodynamic therapy, 35, 102350. doi: 10.1016/j.pdpdt.2021.102350
2	Pourabbas et al.	2023	RCT	Study group: MD + PDT Control group: MD	Peri-implant mucositis	Indocyanine green	–	Diode laser	–	805 nm	0.5 W	–	120 s	1	–	–	Baseline, 2 weeks, and 3 months	ICG-PDT, as an additional therapy to MD, did not lead to any improvements in the clinical or biological parameters of peri-implant mucosal inflammation.	Pourabbas, R., Khorramdel, A., Sadighi, M., Kashefimehr, A., & Mousavi, S. A. (2023). Effect of photodynamic therapy as an adjunctive to mechanical debridement on the nonsurgical treatment of peri-implant mucositis: A randomized controlled clinical trial. Dental research journal, 20, 1.
3	Elsadek et al.	2023	RCT	Study group 1: MD + ICG-PDT Study group 2: MD + MB-PDT Control group: MD	Peri-implantitis (DM2 patients)	Indocyanine green	1 mg/ml	Diode laser	Pulsed mode (100 ms ON/100 ms OFF)	810 nm	300 mW	56 J/cm^2^	30 s	1	–	–	Baseline, 3 and 6 months	Among DM patients with peri-implantitis, adjunctive ICG-PDT and MB-PDT demonstrated comparable outcomes in terms of peri implant clinical and pro-inflammatory characteristics (IL-6 and TNF-α) than MD alone.	Elsadek M. F. (2023). Effectiveness of two photosensitizer-mediated photodynamic therapy for treating moderate peri-implant infections in type-II diabetes mellitus patients: A randomized clinical trial. Photodiagnosis and photodynamic therapy, 43, 103643. doi: 10.1016/j.pdpdt.2023.103643
						Methylene blue	100 μm	Diode laser	Continuous mode	660 ± 10 nm	100 mW	30 J/cm^2^	120 s	1	–	–			
4	Alsayed et al.	2023	RCT	Study group 1: MD + ICG-PDT Study group 2: MD + MB-PDT Control group: MD	Peri-implant mucositis (DM2 patients)	Indocyanine green	1 mg/ml	Diode laser	Continuous mode	810 nm	200 mW	6 J (papilla); 4 J (sulcus)	30 s (papilla); 10 s (sulcus)	1	–	*Fusobacterium nucleatum, Tannerella forsythia, Prevotella intermedia, Porphyromonas gingivalis* and *Aggregatibacter actinomycetemcomitans*	Baseline and 3 months	Compared to conventional MD alone, adjunctive ICG-PDT and MB-PDT resulted in statistically significant improvements in peri-implant clinical, radiographic, microbiological, and immunological parameters at 3-month follow-up among DM2 patients.	Alsayed, H., Bukhari, I. A., Alsaif, R., & Vohra, F. (2023). Efficacy of indocyanine green and methylene blue mediated-photodynamic therapy on peri-implant outcomes among diabetics with peri-implant mucositis. Photodiagnosis and photodynamic therapy, 42, 103344. doi: 10.1016/j.pdpdt.2023.103344
						Methylene blue	0.01%	Diode laser	–	670 nm	140 mW	21 J/cm^2^	–	1	–				
5	Al Rifaiy et al.	2018	RCT	Study group: MD + PDT Control group: MD	Peri-implant mucositis (vaping electronic cigarettes)	Methylene blue	0.005%	Diode laser	–	670 nm	150 mW	–	60 s	1	–	–	3 months	Both groups showed a notable enhancement in PI and PPD at the 12-week follow-up compared to the baseline visit. There was a significant reduction in PI and PPD for PDT as compared to control at 3 months. There was no statistically significant difference for BOP between groups at follow-up.	Al Rifaiy, M. Q., Qutub, O. A., Alasqah, M. N., Al-Sowygh, Z. H., Mokeem, S. A., and Alrahlah, A. (2018). Effectiveness of adjunctive antimicrobial photodynamic therapy in reducing peri-implant inflammatory response in individuals vaping electronic cigarettes: a randomized controlled clinical trial. Photodiagnosis Photodyn. Ther. 22, 132–136. doi: 10.1016/j.pdpdt.2018.03.002
6	ALHarthi et al.	2022	RCT	Study group 1: MD + A single PDT Study group 2: MD + Two repeated PDT Study group 3: MD + Three repeated PDT Control group: MD	Peri-implantitis	Methylene blue	0.005%	Diode laser	–	660 nm	180 mW	0.0125 J/cm^2^ (per site)	60 s	1–3	3 months	–	Baseline, 3, 6 and 9 months	At the 9-month follow-up, PI, GI, and PPD were significantly reduced in all study groups compared to the control group. There was no significant difference in PI, GI and PD in study group 1–3 at 9-months follow-up.	ALHarthi, S. S., Alamry, N. Z., & BinShabaib, M. S. (2022). Effect of multiple sessions of photodynamic therapy on bone regeneration around dental implants among patients with peri-implantitis. Photodiagnosis and photodynamic therapy, 37, 102612. doi: 10.1016/j.pdpdt.2021.102612
7	Shetty et al.	2022	RCT	Study group: MD + PDT Control group: MD	Peri-implant mucositis	Methylene blue	0.005%	Diode laser	–	660 nm	150 mW	–	60 s	1	–	*Oral yeasts*	Baseline and 3 months	At 3-months of follow-up, there was a statistically significant reduction in scores of mPI, mBI, PD and oral yeast colonization among patients in the PDT group compared with the control group.	Shetty, B., Ali, D., Ahmed, S., Ibraheem, W. I., Preethanath, R. S., Vellappally, S., & Divakar, D. D. (2022). Role of antimicrobial photodynamic therapy in reducing subgingival oral yeasts colonization in patients with peri-implant mucositis. Photodiagnosis and photodynamic therapy, 38, 102803. doi: 10.1016/j.pdpdt.2022.102803
8	Bassetti et al.	2014	RCT	Study group: MD + PDT Control group: MD + LDD	Peri-implantitis	Phenothiazine chloride	–	Diode laser	–	660 nm	100 mW	–	10 s	2	1 week	*Porphyromonas gingivalis (P.g), Tannerella forsythia (T.f), Treponema denticola, Aggregatibacter actinomycetemcomitans, Prevotella intermedia, Campylobacter rectus, Fusobacterium nucleatum, Capnocytophaga gingivalis, Parvimonas micra, Eubacterium nodatum,* and *Eikenella corrodens*	3, 6, 9, and 12 months	Compared to the baseline, PPD significantly declined at PDT-treated areas for up to 9 months and at LDD-treated areas for up to 12 months. Counts of *Porphyromonas gingivalis* and *Tannerella forsythia* decreased statistically significantly from baseline to 6 months in the PDT and to 12 months in the LDD group, respectively. A significant decrease in CF IL-1b levels was observed in both groups from baseline to 12 months. There were no significant differences in clinical, microbiological, and host-derived parameters between the groups after 12 months.	Bassetti, M., Schär, D., Wicki, B., Eick, S., Ramseier, C. A., Arweiler, N. B., et al. (2014). Anti-infective therapy of peri-implantitis with adjunctive local drug delivery or photodynamic therapy: 12-month outcomes of a randomized controlled clinical trial. Clin. Oral Implants Res. 25, 279–287. doi: 10.1111/clr.12155
9	Javed et al.	2017	RCT	Study group: MC + PDT Control group: MC	Peri-implant mucositis	Phenothiazine chloride	–	Diode laser	–	660 nm	100 mW	–	10 s	1	–	–	3 months	Periimplant PI, BOP and PPD were comparable in both groups at baseline. At 12-weeks, there was a significant reduction in PI and PPD among patients in both two groups compared with their respective baselines. At 12-weeks, PI and PPD were significantly higher among patients in PDT group compared with control group BOP was comparable in both groups at baseline and at 12-weeks.	Javed, F., Bin Shabaib, M. S., Alharthi, S. S., and Qadri, T. (2017). Role of mechanical curettage with and without adjunct antimicrobial photodynamic therapy in the treatment of peri-implant mucositis in cigarette smokers: a randomized controlled clinical trial. Photodiagnosis Photodyn. Ther. 18, 331–334. doi: 10.1016/j.pdpdt.2017.04.015
10	De Angelis et al.	2012	RCT	Study group: MD + PDT Control group: MD	Peri-impactites	Tolouidine blue O	0.1 mg/ml	LED	–	630 nm	–	–	80 s	1	–	–	4 months	The application of PDT in conjunction with mechanical cleaning for implants with peri-implantitis did not yield any enhanced clinical outcomes to 4 months of treatment.	De Angelis, N., Felice, P., Grusovin, M. G., Camurati, A., and Esposito, M. (2012). The effectiveness of adjunctive light-activated disinfection (LAD) in the treatment of peri-implantitis: 4-month results from a multicentre pragmatic randomised controlled trial. Eur. J. Oral Implantol. 5, 321–331.
11	Karimi et al.	2016	RCT	Study group: MD + PDT Control group: MD	Peri-impactites and peri-implant mucositis	Toluidine blue	0.01%	LED	–	630 nm	2,000 mW/cm^2^	–	20 s (per site); 2 min (total)	1	–	–	1.5 and 3 months	Statistical analysis showed significant differences in PPD, CAL, BOP, and GI at each time point between the two groups. There were no statistically significant changes with respect to any of the parameters in the control group. Complete resolution of BOP at 3 months was achieved in 100% of test implants. At 1.5 and 3 months, there were significant differences in the mean probing depth and CAL gain measurements at implants in the study group.	Karimi, M. R., Hasani, A., and Khosroshahian, S. (2016). Efficacy of antimicrobial photodynamic therapy as an adjunctive to mechanical debridement in the treatment of Peri-implant diseases: a randomized controlled clinical trial. J. Lasers Med. Sci. 7, 139–145. doi: 10.15171/jlms.2016.24
12	Zeza et al.	2018	RCT	Study group: PAPR + PDT	Peri-implant mucositis	Toluidine blue O	–	LED	–	630 nm	–	–	10 s	1	–	–	2 and 6 weeks	The results of the study indicate that treatment with PAPR and PDT resulted in a significant reduction in the median number of BoP + sites from 1 to 0 around teeth and from 3.5 to 2.0 around implants.	Zeza, B., Farina, R., Pilloni, A., and Mongardini, C. (2018). Clinical outcomes of experimental gingivitis and peri-implant mucositis treatment with professionally administered plaque removal and photodynamic therapy. Int. J. Dent. Hyg. 16, e58–e64. doi: 10.1111/idh.12302

BOP, bleeding on probing; CAL, clinical attachment loss; cm^2^, square centimeter; DM2, type 2 Diabetes; GI, gingival index; ICG, indocyanine green; LDD, local drug delivery; MB, methylene blue; MC, mechanical curettage; MD, mechanical debridement; min, minutes; mW, milliwatts; nm, nanometers; μm, micrometer; PAPR, professionally administered plaque removal; PCBL, peri implant crestal bone loss; PI, plaque index; PIMD, peri-implant manual debridement; PPD, pocket probing depth; RCT, randomized clinical trial; s, seconds; –, not reported.

For nonsurgical peri-implantitis treatment, adjunctive use of PDT has been investigated in four randomized controlled studies (47–50), and all studies have shown significant improvements for PD, BOP, and CBL. As for bacteria count measurement, Labban et al. (51) found that PDT application significantly reduced peri-implant pathogens than mechanical debridement. Oral yeasts are also related to the etiopathogeneses of peri-implant disorders besides bacteria (52). In patients with peri-implant mucositis, Shetty et al. (53) investigated the effectiveness of 0.005% methylene-blue with 660 nm diode laser at the energy density of 16.8 J/cm^2^ in lowering subgingival oral yeast colonization (OYC). After a 3-month follow-up, the modified plaque index (mPI), modified bleeding index (mBI), PD, and OYC scores in the PDT group were lower than those in the control group.

The *in vitro* investigation proved that *T. Forsythia* and *P. gingivalis* grown on titanium specimens subjected to PDT mediated by 312 µM methylene blue for 1 min under a 685 nm diode laser with a dosage of 7.9 J/cm^2^ could significantly lower *T. forsythia* and *P. gingivalis* biofilm compared with neodymium-doped yttrium aluminum garnet (Nd: YAG) laser, H_2_O_2_, and chlorhexidine groups (54). The study also revealed that surface roughness remained constant while the contact angle decreased in the PDT group. In another *vitro* investigation, Anil et al. (55) discovered that methylene blue-PDT significantly reduced *P. gingivalis* and *T. Forsythia* viability over Zirconia specimens in comparison with other disinfection groups (the hydrogen peroxide group, the Nd: YAG laser, and the chlorhexidine group). The PDT approach decreased contact angles from zirconia specimens, suggesting increased hydrophilicity. PDT had the highest surface free energy (SFE) score (41.68) across all decontamination methods, followed by chlorhexidine (39.83), Nd: YAG (34.52), and H_2_O_2_ (29.88).

## 7 Endodontic root canal infections

Pulpitis and periapical periodontitis are prevalent oral disorders mostly attributed to anaerobic bacteria, including *P. gingivalis* and *Enterococcus faecalis (E. faecalis)*. *E. faecalis* is a frequent species of re-infection after root canal therapy, owing to factors such as antibiotic resistance, microbial biofilm formation, and dentinal penetration, whose eradication poses significant challenges (56). Although the main treatment for pulpitis and periapical periodontitis is root canal therapy, the complexity of the root canal system (communicating branches, lateral branches, and an apical bifurcation) and the multispecies biofilm communities have made the microbial biofilm complete removal more challenging. Clinical studies on this topic recommend that PDT could be a promising technique to eliminate root canal bacteria after standard chemo mechanical debridement (Table 6).

**Table 6:** Summary of studies on PDT for the treatment of endodontic infections.

	Study	Year	Study design	Treatment groups	Lesion type	Type of photosensitizers	Photosensitizers dose	Light source	Irradiation mode	Wave length	Fluence rate/Power	Energy density	Irradiation time	Treatment sessions	Treatment interval	Microorganisms	Follow up	Primary outcome	Referrence
1	Ahangari et al.	2017	RCT	Group 1: CMD + PDT Group 2: CMD + Calcium hydroxide therapy	Persistent endodontic infection	Methylene blue	0.05 mg/ml	Diode laser	–	810 nm	200 mW	–	10 s	1	–	*Enterococcus faecalis* and *Candida albicans*	2 weeks	Number of CFU significantly decreased in both groups after the interventions; however, there was no significant difference in the colony count between the 2 groups.	Ahangari, Z., Mojtahed Bidabadi, M., Asnaashari, M., Rahmati, A., and Tabatabaei, F. S. (2017). Comparison of the antimicrobial efficacy of calcium hydroxide and photodynamic therapy against enterococcus faecalis and Candida albicans in teeth with Periapical lesions; an in vivo study. J Lasers Med. Sci. 8, 72–78. doi: 10.15171/jlms.2017.13
2	da Silva et al.	2018	RCT	Study group: CMD + PDT Control group: CMD	Primary endodontic infection	Methylene blue	0.1 mg/ml	Diode laser	–	660 nm	100 mW	3 J	30 s	1	–	*Enterococcus faecalis, Candida genus* and *Bacteria domain*	1 week	PDT significantly reduced the incidence of *E. faecalis* before root canal obturation at the second session in teeth with primary endodontic infections.	da Silva, C. C., Chaves Júnior, S. P., Pereira, G. L. D., Fontes, K., Antunes, L. A. A., Póvoa, H. C. C., et al. (2018). Antimicrobial photodynamic therapy associated with conventional endodontic treatment: a clinical and molecular microbiological study. Photochem. Photobiol. 94, 351–356. doi: 10.1111/php.12869
3	de Miranda and Colombo	2018	RCT	Study group: CMD + PDT Control group: CMD	Persistent endodontic infection	Methylene blue	25 μg/ml	Diode laser	–	660 nm	100 mW	–	5 min	1	–	*Candida albicans, Dialister pneumosintes, Prevotella nigrescens, Prevotella tannerae, Parvimonas micra, Peptostreptococcus anaerobius, Propionibacterium acnes,* and *others*	3 and 6 months	Traditional endodontic treatment, whether combined with PDT or not, effectively decreases microbial presence, leading to periapical recovery. However, adjunctive PDT leads to improved periapical healing at 6-month follow-up.	de Miranda, R. G., and Colombo, A. P. V. (2018). Clinical and microbiological effectiveness of photodynamic therapy on primary endodontic infections: a 6-month randomized clinical trial. Clin. Oral Investig. 22, 1751–1761. doi: 10.1007/s00784-017-2270-4
4	Coelho et al.	2019	RCT	Study group: CT + PDT Control group: CT	Primary endodontic infection	Methylene blue	1.56 μm/ml	Diode laser	–	660 nm	100 mW	600 J/cm^2^	3 min	1	–	–	24 h, 72 h and 1 week	PDT significantly reduced post-operative pain at 24 and 72 hours in the treatment of single-rooted teeth with necrotic pulps completed in one visit.	Coelho, M. S., Vilas-Boas, L., and Tawil, P. Z. (2019). The effects of photodynamic therapy on postoperative pain in teeth with necrotic pulps. Photodiagnosis Photodyn. Ther. 27, 396–401. doi: 10.1016/j.pdpdt.2019.07.002
5	Okamoto et al.	2020	RCT	Study group: CT + PDT Control group: CT	Primary endodontic infection	Methylene blue	0.005%	–	–	660 nm	100 mW	4 J/cm^2^	40 s	1	–	Total viable bacteria load	1 and 3 months	The reduction in bacterial load was 93% in control group and 99% in PDT group, with no statistically significant difference.	Okamoto, C. B., Bussadori, S. K., Prates, R. A., da Mota, A. C. C., Tempestini Horliana, A. C. R., Fernandes, K. P. S., et al. (2020). Photodynamic therapy for endodontic treatment of primary teeth: a randomized controlled clinical trial. Photodiagnosis Photodyn. Ther. 30:101732. doi: 10.1016/j.pdpdt.2020.101732
6	Guimaraes et al.	2021	RCT	Study group: CT + PDT + LLLT Control group: CT	Primary endodontic infection	Methylene blue	0.01%	Diode laser	–	660 nm	100 mW	300 J/cm^2^	90 s	1	–	–	2, 3 and 7 days	At any observation time, there were no significant disparities in post-operative pain, tenderness, oedema, or the use of analgesics between the groups.	Guimaraes, L. D. S., da Silva, E. A. B., Hespanhol, F. G., Fontes, K. B. F. D. C., Antunes, L. A. A., and Antunes, L. S. (2021). Effect of photobiomodulation on post operative symptoms in teeth with asymptomatic apical periodontitis treated with foraminal enlargement: a randomized clinical trial. Int. Endod. J. 54, 1708–1719. doi: 10.1111/iej.13593
7	Moreira et al.	2021	RCT	Study group: CT + intracanal medication + PDT Control group: CT + intracanal medication	Primary endodontic infection	Methylene blue	0.005%	Laser Duo device	–	660 nm	–	–	90 s	2	15 days	*Enterococcus faecalis* and *Actinomyces israelii*	2 months	PDT did not promote better results in endodontic treatment, in comparison with conventional treatment.	Moreira, S. D. A., Nunes, J. B., Colombo, F. A., Fonseca, N. D. S. M., and Viola, N. V. (2021). Radiographic and antimicrobial evaluation of enterococcus Faecalis and Actinomyces Israelii micro-organisms after photodynamic therapy (aPDT). Photodiagnosis Photodyn. Ther. 35:102433. doi: 10.1016/j.pdpdt.2021.102433
8	Alves-Silva et al.	2022	RCT	Study group: CT + PDT Control group: CT	Primary endodontic infection	Methylene blue	0.005%	Diode laser	–	660 nm	100 mW	320 J/cm^2^	90 s	1	–	–	8, 12, 24, 48, 72 h and 1 week	There was a statistically significant difference in the periods of 8, 12, 24, 48 and 72 h between the control group and the PDT group. After 1 week, there was no statistically significant difference.	Alves-Silva, E. G., Arruda-Vasconcelos, R., Louzada, L. M., De-Jesus-Soares, A., Ferraz, C. C. R., Almeida, J. F. A., et al. (2022). The effect of photodynamic therapy on postoperative pain in teeth with primary endodontic infection. Photodiagnosis Photodyn. Ther. 37:102700. doi: 10.1016/j.pdpdt.2021.102700
9	Asnaashari et al.	2017	RCT	Group 1: CT + PDT Group 2: CT + Calcium hydroxide therapy	Persistent endodontic infection	Tolouidine blue O	0.1 mg/ml	LED	–	635 nm	2–4 mW/cm^2^	–	60 s	1	–	*Enterococcus faecalis*	2 weeks	PDT leads to a greater reduction in *enterococcus faecalis* number compared with calcium hydroxide therapy.	Asnaashari, M., Ashraf, H., Rahmati, A., and Amini, N. (2017). A comparison between effect of photodynamic therapy by LED and calcium hydroxide therapy for root canal disinfection against enterococcus faecalis: a randomized controlled trial. Photodiagnosis Photodyn. Ther. 17, 226–232. doi: 10.1016/j.pdpdt.2016.12.009
10	Di Taranto et al.	2022	RCT	Group 1: CT + high-power laser Group 2: CT + PDT	Primary endodontic infection	Tolouidine blue O	155 μg/ml	Diode laser	–	660 nm	100 mW	–	–	1	–	*Enterococcus sp., Candida sp., Lactobacillus sp.* and *Phorphyromonas sp*	1 week	The photodynamic laser treatment tested yields positive and clinically significant outcomes when used alongside traditional mechanical and chemical cleaning. There is a further reduction after a second treatment with photodynamic therapy.	Di Taranto, V., Libonati, A., Montemurro, E., Gallusi, G., and Campanella, V. (2022). Antimicrobial effects of photodynamic and high-power laser endodontic therapy on patients with necrotic pulp and periapical lesion. J. Biol. Regul. Homeost. Agents 36, 41–48.

cm^2^, square centimeter; min, minutes; CMD, chemo-mechanical debridement; CT, conventional endodontic therapy; LLLT, low-level laser therapy; mW, milliwatts; nm, nanometers; RCT, randomized clinical trial; s, seconds; –, not reported.

Alves-Silva et al. (57) evaluated the effectiveness of PDT as an additional treatment for improving bacterial clearance and reducing lipopolysaccharide (LPS) and lipoteichoic acid (LTA) levels. The study involved two groups: one group received endodontic therapy with chemo-mechanical preparation (CMP) alone, while the other group received PDT (using a 9 J/cm^2^ 660 nm red laser with 0.005% methylene blue for 3 min) following CMP. The results revealed that root canals had samples with pulp necrosis and periapical lesions. Additionally, LPS and LTA levels were significantly reduced in the PDT group, with a higher reduction in the CMP group.

Pourhajibagher et al. performed a comprehensive study and synthesis of existing studies to examine the effectiveness of combining PDT with standard chemo-mechanical debridement in treating infected root canal systems in endodontic diseases (58). Their research revealed a substantial decrease in the amount of microorganisms when PDT was used in addition to other treatments.

## 8 Oral fungal infections

Candida species are the most common fungi indigenous to human mucosal surfaces, which is mainly caused by *Candida albicans* (*C. albicans*). Although *C. albicans* is a commensal organism in healthy individuals, it could convert into a pathogenic one following changes in the host environment. *Oral candidiasis* (OC) is the most commonly encountered oral manifestation, which is mainly caused by *C. albicans* infection (59). Traditional antifungal treatment applied in OC include amphotericin B, nystatin, clotrimazole and ketoconazole (60, 61). However, the limitation of antifungal drugs, high resistance and host defense mechanisms made antifungal treatment difficult. Hence, alternative strategies such as PDT against the emergence of drug-resistant *C. albicans* are being considered (Table 7).

**Table 7:** Summary of studies on PDT for the treatment of oral fungal infections.

	Study	Year	Study design	Treatment groups	Lesion type	Type of photosensitizers	Photosensitizers dose	Light source	Irradiation mode	Wave length	Fluence rate/Power	Energy density	Irradiation time	Treatment sessions	Treatment interval	Microorganisms	Follow up	Primary outcome	Referrence
1	Scwingel et al.	2012	RCT	Study group 1: LLLT Study group 2: PDT Control group: 100 mg/day Fluconazole (14 days)	Oral candidiasis (HIV-Infected Patients)	Methylene blue	450 μg/ml	Twin Laser	–	660 nm	30 mW	7.5 J/cm^2^	10 s/point	1	–	*Candida spp.*	Immediately, and 7, 15, and 30 days	PDT eradicated 100% colonies of this fungus, and the patients showed no recurrence of candidiasis for up to 30 days after radiation.	Scwingel, A. R., Barcessat, A. R. P., Núnez, S. C., and Ribeiro, M. S. (2012). Antimicrobial photodynamic therapy in the treatment of oral candidiasis in HIV-infected patients. Photomed. Laser Surg. 30, 429–432. doi: 10.1089/pho.2012.3225
2	Maciel et al.	2016	RCT	Study group: PDT + LLLT Control group: Oral miconazole gel	Denture stomatitis	Methylene blue	0.01%	Diode laser	–	660 nm	100 mW/cm^2^	1 J/cm^2^	10 s	1	–	*Candida spp.*	1 month	After fifteen days from the end of treatment, the recurrence rate was 25% in patients who received PDT with LLLT therapy, compared to 12.5% in those treated with miconazole.	Maciel, C. M., Piva, M. R., Ribeiro, M. A. G., de Santana Santos, T., Ribeiro, C. F., and Martins-Filho, P. R. S. (2016). Methylene blue-mediated photodynamic inactivation followed by low-laser therapy versus Miconazole gel in the treatment of denture stomatitis. J. Prosthodont. 25, 28–32. doi: 10.1111/jopr.12284
3	de Senna et al.	2018	RCT	Study group: PDT Control group: oral miconazole gel	Denture stomatitis	Methylene blue	450 μg/ml	Diode laser	–	660 nm	100 mW	28 J/cm^2^	–	8	Twice a week	*C. albicans C. tropicalis C. glabrata*	7, 15 and 30 days	While PDT was statistically more effective in alleviating inflammation after 15 days, the difference was not significant after 30 days.	de Senna, A. M., Vieira, M. M. F., Machado-de-Sena, R. M., Bertolin, A. O., Núñez, S. C., and Ribeiro, M. S. (2018). Photodynamic inactivation of Candida ssp. on denture stomatitis. A clinical trial involving palatal mucosa and prosthesis disinfection. Photodiagnosis Photodyn. Ther. 22, 212–216. doi: 10.1016/j.pdpdt.2018.04.008
4	Alrabiah et al.	2019	RCT	Study group: PDT Control group: Topical nystatin	Denture stomatitis	Methylene blue	450 μg/ml	Diode laser	Continuous mode	660 nm	100 mW	28 J/cm^2^	–	8	Twice a week	*C. albicans C. tropicalis C. glabrata*	1 and 2 months	Both groups led to a significant reduction in the number of *C. albicans*, but the difference between them was not notable.	Alrabiah, M., Alsahhaf, A., Alofi, R. S., Al-Aali, K. A., Abduljabbar, T., and Vohra, F. (2019). Efficacy of photodynamic therapy versus local nystatin in the treatment of denture stomatitis: a randomized clinical study. Photodiagnosis Photodyn. Ther. 28, 98–101. doi: 10.1016/j.pdpdt.2019.08.028
5	Fonseca et al.	2022	RCT	Group 1: MB-PDT Group 2: CUR-PDT	Oral candidiasis (patients with head and neck cancer)	Methylene blue	300 µmol/L	Red laser	–	660 nm	–	300 J/cm^2^	90 s	4	Twice a week	*Strains of C. tropicalis, C. parapsilosis, C. krusei,* and *C. glabrata*	2 weeks	There was no difference in treatment failure evaluated by the necessity of drug prescription. MB-PDT reduced the number of infected anatomical sites compared to CUR-PDT. Between the groups, no differences were found in the DNA quantification of *Candida sp*.	Fonseca, L. L., Durães, C. P., Menezes, A. S. D. S., Tabosa, A. T. L., Barbosa, C. U., Filho, A. P. S., Souza, D. P. S. P., Guimarães, V. H. D., Santos, S. H. S., de Paula, A. M. B., Farias, L. C., & Guimarães, A. L. S. (2022). Comparison between two antimicrobial photodynamic therapy protocols for oral candidiasis in patients undergoing treatment for head and neck cancer: A two-arm, single-blind clinical trial. Photodiagnosis and photodynamic therapy, 39, 102983. doi: 10.1016/j.pdpdt.2022.102983
						Curcumin	80 µmol/L	LED	–	480 nm	–	200 J/cm^2^	90 s						
6	de Souto Medeiros et al.	2023	RCT	Study group: PDT Control group: Nystatin	Oral erythematous candidiasis	Methylene blue	0.1%	MM Optics laser	Continuous mode	660 nm	–	4 J (per point)	40 s (per point)	≤4	1 week	*Candida* and *Staphylococcus sp.*	Weekly follow-up until end of treatment	Complete remission of the lesions was observed in 16 (94.1%) of the control group and 16 (84.2%) of the PDT group. It was noted that severe lesions were harder to present remission, whereas all mild and moderate lesions completely regressed.	de Souto Medeiros, M. R., da Silva Barros, C. C., de Macedo Andrade, A. C., de Lima, K. C., & da Silveira, É. J. D. (2023). Antimicrobial photodynamic therapy in the treatment of oral erythematous candidiasis: a controlled and randomized clinical trial. Clinical oral investigations, 27(11), 6471–6482. doi: 10.1007/s00784-023-05252-3
7	Al-Aali et al.	2023	RCT	Group 1: Miconazole Group 2: PDT Group 3: Miconazole + PDT Group 4: Chlorhexidine Group 5: Distilled water	Denture stomatitis (DM2 patients)	Methylene blue	0.005%	Diode laser	–	660 nm	3527 mW/cm^2^	9 J	–	1	–	*Candida spp.*	Baseline, end of 14 days, 28 days and 60 days	In the group that received combination treatment, there was a notable enhancement in quality of life. Significant differences in CFU/mL values were observed in the combination treatment group during all study periods.	Al-Aali, K. A., Alqahtani, A. S., AlZaid, A. A., Almujel, S. H., Alsaloum, M., & Alanazi, K. K. (2023). Efficacy of adjunct photodynamic therapy on Candida growth and oral health quality of life in denture stomatitis patients with type 2 diabetes mellitus wearing implant-retained overdentures: A randomized clinical study. Photodiagnosis and photodynamic therapy, 42, 103630. doi: 10.1016/j.pdpdt.2023.103630
8	Labbans et al.	2021	RCT	Group 1: RB-PDT Group 2: CUR-PDT Group 3: Nystatin	Denture stomatitis (patients with habitual cigarette smoking)	Rose Bengal or Curcumin	0.8 μg/ml	LED	–	440–460 nm	102 mW/cm^2^ (palate); 24 mW/cm^2^ (denture)	122 J/cm^2^ (palate); 37.5 J/cm^2^ (denture)	20 min (palate); 26 min (denture)	6	Thrice per week	*C. albicans, C. tropicalis,* and *C. glabrata*	Baseline, 6 weeks and 12 weeks	PDT using CUR and RB was as effective as topical Nystatin treatment for managing denture stomatitis in cigarette smokers.	Labban, N., Taweel, S. M. A., ALRabiah, M. A., Alfouzan, A. F., Alshiddi, I. F., & Assery, M. K. (2021). Efficacy of Rose Bengal and Curcumin mediated photodynamic therapy for the treatment of denture stomatitis in patients with habitual cigarette smoking: A randomized controlled clinical trial. Photodiagnosis and photodynamic therapy, 35, 102380. doi: 10.1016/j.pdpdt.2021.102380
9	de Cássia Dias Viana Andrade al.	2022	RCT	Study group 1: LLLT Study group 2: PDT Control group: Nystatin	Oral mucositis in oncologic patients	Curcumin	0.75 mg/ml	LED	–	450 nm	67 mW/cm^2^	20.1 J/cm^2^	10 min	4	1 week	*Candida spp.*	7, 14, 21 and 30 days	PDT showed a greater reduction of yeasts of the genus *Candida* in the tested parameters.	de Cássia Dias Viana Andrade, R., Azevedo Reis, T., Rosa, L. P., de Oliveira Santos, G. P., & da CristinaSilva, F. (2022). Comparative randomized trial study about the efficacy of photobiomodulation and curcumin antimicrobial photodynamic therapy as a coadjuvant treatment of oral mucositis in oncologic patients: antimicrobial, analgesic, and degree alteration effect. Supportive care in cancer: official journal of the Multinational Association of Supportive Care in Cancer, 30(9), 7365–7371. doi: 10.1007/s00520-022-07127-x
10	Afroozi et al.	2019	RCT	Study group: PDT + Nystatin Control group: Nystatin	Denture stomatitis	Indocyanine green	1 mg/ml	Diode laser	Continuous mode	810 nm	–	56 J/cm^2^	30 s	2	1 week	*Candida spp*	2 months	The combination of PDT and nystatin resulted in a significantly greater mean reduction than nystatin alone.	Afroozi, B., Zomorodian, K., Lavaee, F., Zare Shahrabadi, Z., and Mardani, M. (2019). Comparison of the efficacy of indocyanine green-mediated photodynamic therapy and nystatin therapy in treatment of denture stomatitis. Photodiagnosis Photodyn. Ther. 27, 193–197. doi: 10.1016/j.pdpdt.2019.06.005
11	Mima et al.	2012	RCT	Study group: PDT Control group: Topical nystatin	Denture stomatitis	Hematoporphyrin derivative	500 mg/L	LED	–	455 nm	24 mW/cm^2^	37.5, 122 J/cm^2^	20, 26 min	6	–	*C. albicans, C. tropicalis* and *C. glabrata*	1, 2, and 3 months	Both groups led to a significant reduction in CFU/mL at the end of the treatments and on day 30 of the follow-up period. The Nystatin and PDT groups showed clinical success rates of 53% and 45%, respectively.	Mima, E. G., Vergani, C. E., Machado, A. L., Massucato, E. M. S., Colombo, A. L., Bagnato, V. S., et al. (2012). Comparison of photodynamic therapy versus conventional antifungal therapy for the treatment of denture stomatitis: a randomized clinical trial. Clin. Microbiol. Infect. 18, E380–E388. doi: 10.1111/j.1469-0691.2012.03933.x
12	Alves et al.	2020	RCT	Study group: PDT Control group: Topical nystatin	Denture stomatitis	Photodithazine	200 mg/L	LED	–	660 nm (peak)	240 mW/cm^2^ (palate); 50 mW/cm^2^ (denture)	50 J/cm^2^	4 min (palate); 17 min (denture)	6	Three times a week	*C. albicans, C. tropicalis* and *C. glabrata*	15, 30 and 45 days	PDT was more effective to reduce the Candida spp. than nystatin. However, both groups showed recurrence.	Alves, F., Carmello, J. C., Alonso, G. C., Mima, E. G. D. O., Bagnato, V. S., and Pavarina, A. C. (2020). A randomized clinical trial evaluating Photodithazine-mediated antimicrobial photodynamic therapy as a treatment for denture stomatitis. Photodiagnosis Photodyn. Ther. 32:102041. doi: 10.1016/j.pdpdt.2020.102041

cm^2^, square centimeter; CUR, curcumin; DM2, type 2 Diabetes; LLLT, low-level laser therapy; min, minutes; mW, milliwatts; MB, methylene blue; nm, nanometers; μm, micrometer; RB, Rose Bengal; RCT, randomized clinical trial; s, seconds; TBO, toluidine blue O; –, not reported.

Ma et al. (62) conducted investigations on the impact of curcumin on biofilms of *C. albicans*. The researchers carried out experimental analyses on a standard strain as well as two clinical isolates obtained from individuals with HIV and oral lichen planus. The findings of their study indicated that a 20 min pre-irradiation of 60 µM curcumin and a 6 min exposure to LED with a dosage of 7.92 J/cm^2^ resulted in a reduction of *C. albicans* biofilms. Furthermore, expression of efg1, ume6, hgc1, and ece1 genes expression of *C. albicans* was decreased after PDT treatment. Additionally, ALA–PDT (using a 635 nm red laser at 300 J/cm^2^) showed potent inhibition of the metabolic activity of *C. albicans* (63). Pereira et al. (64) evaluated potential effects of 200 µM erythrosine plus a 532 ± 10 nm green LED (237 mW/cm^2^, 42.63 J/cm^2^) in planktonic culture, biofilms and virulence factors of *Candida* strains. The results indicated that the addition of PDT could significantly reduce Candida species growth as well as lower the virulence and pathogenicity of certain Candida species, however, there was a greater resistance to PDT in biofilm structures compared to planktonic cultures.

Hu et al. (65) conducted a meta-analysis of 11 trials to assess the impact of PDT as a supplementary or substitute treatment for oral candidiasis compared to conventional antifungal medications such as nystatin, fluconazole, and miconazole. The results demonstrated that PDT outperformed nystatin in reducing the number of oral candida colonies in the palates of patients. However, no statistically significant difference was observed in the denture location. Fluconazole and PDT had similar efficiency in the treatment of oral candidiasis. However, miconazole was shown to be more effective than PDT. However, the use of PDT in conjunction with nystatin has been found to be a more effective treatment for oral candidiasis, with improved effectiveness and a lower chance of the condition recurrence.

## 9 Perspectives and future directions

### 9.1 Recent clinical advances in PDT

PDT development direction mainly focuses on development of photosensitizer and laser development and utilization to enhance its targeting and light penetration ability. Combining functionalized nano-materials, radiosensitizers, and hyperthermia with PDT can enhance the synergistic effect of photosensitizers and increase the curative effect on deep tissues. Numerous functional nano-materials have been created due to the quick development of nanotechnology for better medication delivery and anticancer and antibacterial effects. Many different nanoparticles have been created, including 5-ALA-loaded chitosan-tripolyphosphate nanoparticles (CS-TPP NPs) (66), MPP (Polymers) combined with Ce6 (67), and rose bengal (RB) in silver nanoclusters (AgNCs) (68), among others (69). Sonodynamic therapy employs ultrasonic waves to activate photosensitizers, inducing singlet oxygen production. This mechanism serves as a compensatory strategy for the limited tissue penetration capabilities of light-based therapies (70). Photochemical internalization (PCI) is a new method wherein photosensitizers and cytotoxic substances (including bleomycin) are injected into tissues. Subsequently, the cytotoxic molecules exert their effects on the cytoplasm through light internalization. Two-photon absorption PDT uses two wavelengths of photons to reach the photosensitizer simultaneously to increase the absorption power and intensity to act on cells. This brings a new research direction for PDT, makes up for the shortcomings of traditional PDT, and hopefully becomes a new method widely used in clinical treatment. Aiming at increasing the therapeutic efficiency, combination regimens through multiple photosensitizers with multiple certain wavelengths of light sources either sequentially or simultaneously, is needed in the future.

### 9.2 Limitations

PDT is ROS-dependent, non-invading and convenient. However, all three crucial elements for PDT (PS, light, and oxygen) could contribute to the limitation of ROS generation. Poor tissue penetration occurs with short wavelength lights. Due to the hypoxic tumor environment caused by extensive areas of tumoral necrosis and local hypoxia, which results in a poor response to PDT, the absolute demand for oxygen may be insufficient. The severe limitations of conventional PS, including poor solubility, low stability, and inadequate tissue penetration, continue to be a barrier to PDT, requiring innovative solutions to improve PDT clinical results.

### 9.3 Current controversies in PDT

Before PDT becomes widely used in therapeutic settings, various questions about its utilization must be cleared up; PDT toxicity, oral ecosystem balance, and impact of complicated infection should be investigated *in vitro* and *in vivo* research. Photosensitizers, including MB, toluidine blue O (TBO), or malachite green, can alter tooth structure shade. More PS molecules can permeate the internal composition of the tooth with the increased PS incubation time.

## 10 Conclusions

Although PDT has demonstrated much promise and usefulness in dentistry, it is important to acknowledge several limitations. These factors encompass the restricted capacity of the PS to deeply enter dentinal tubules, difficulties in the transmission of light, and the lack of oxygen in deep periodontal pockets. Moreover, there is a lack of consensus about the most effective treatment strategy for oral problems. Therefore, further thorough and carefully controlled study is necessary to determine the most efficient PS, the suitable irradiation protocols, and the optimal wavelengths for activating the PS. This will enable healthcare practitioners to achieve the intended outcomes.
